# Hybrid multimodule DC–DC converters accelerated by wide bandgap devices for electric vehicle systems

**DOI:** 10.1038/s41598-024-55426-6

**Published:** 2024-02-27

**Authors:** Abdul Waheed, Saif ur Rehman, Faisal Alsaif, Shoaib Rauf, Ismail Hossain, Mukesh Pushkarna, Fsaha Mebrahtu Gebru

**Affiliations:** 1 Department of Electrical Engineering, The Superior University, Lahore, 54000 Pakistan; 2 Department of Electrical Engineering, College of Engineering, King Saud University, 11421 Riyadh, Saudi Arabia; 3 Department of Electrical Engineering, University of Gujrat, Gujrat, Pakistan; 4 Department of Nuclear and Renewable Energy, Ural Federal University, 620002 Yekaterinburg, Russia; 5 Department of Electrical Engineering, GLA University Mathura, Mathura, India; 6 Department of Electrical and Computer Engineering, Raya University, Raya, Ethiopia

**Keywords:** Binary genetic algorithm, Principal component analysis, Mean absolute percentage error, Load forecasting, Energy science and technology, Engineering, Mathematics and computing

## Abstract

In response to the growing demand for fast-charging electric vehicles (EVs), this study presents a novel hybrid multimodule DC–DC converter based on the dual-active bridge (DAB) topology. The converter comprises eight modules divided into two groups: four Insulated-Gate Bipolar Transistor (IGBT) modules and four Metal–Semiconductor Field-Effect Transistor (MESFET) modules. The former handles high power with a low switching frequency, while the latter caters to lower power with a high switching frequency. This configuration leverages the strengths of both types of semiconductors, enhancing the converter’s power efficiency and density. To investigate the converter’s performance, a small-signal model is developed, alongside a control strategy to ensure uniform power sharing among the modules. The model is evaluated through simulation using MATLAB, which confirms the uniformity of the charging current provided to EV batteries. The results show an impressive power efficiency of 99.25% and a power density of 10.99 kW/L, achieved through the utilization of fast-switching MESFETs and the DAB topology. This research suggests that the hybrid multimodule DC–DC converter is a promising solution for fast-charging EVs, providing high efficiency, power density, and switching speed. Future studies could explore the incorporation of advanced wide bandgap devices to handle even larger power fractions.

## Introduction

Fossil fuels have historically served as the world’s primary energy source, offering numerous benefits. However, they also pose significant challenges, including the release of harmful emissions that contribute to environmental degradation. Moreover, the diminishing availability of fossil fuel reserves is a growing concern, with potentially severe consequences for energy security and sustainability. Recognizing these issues, the scientific community and policymakers have increasingly focused on developing alternative, renewable energy sources. Renewable energy, which includes solar, wind, geothermal, and hydroelectric power, has emerged as a viable and sustainable solution to address the shortcomings of conventional fossil fuel-based energy systems^1^. Solar power, in particular, has tremendous potential, with the Earth receiving an astounding 121,800 terawatts of solar energy per hour. This vast and virtually inexhaustible resource presents an opportunity to meet global energy needs in a more environmentally friendly and sustainable manner. The declining cost of photovoltaic (PV) technology has made solar power increasingly competitive, accelerating its adoption in the production of clean, green energy^2^. Electric vehicles (EVs) represent another crucial aspect of the transition to renewable energy. EVs, which have a long history dating back to 1868, offer an alternative to gasoline-powered vehicles that produce harmful emissions. Integrating EVs into the distribution grid with EV charging stations is a key step toward reducing our reliance on fossil fuels and mitigating the environmental impact of transportation^3^. In conclusion, renewable energy, particularly solar power, holds tremendous promise for a sustainable future. By embracing green energy technologies like PV and EVs, we can mitigate the environmental impact of energy generation and consumption, reduce our dependence on finite fossil fuel reserves, and pave the way for a cleaner, greener planet. Due to various drawbacks such as cost, less range, and a lack of charging infrastructure, electric vehicles present in the market possess less share. The researchers are researching how various bottlenecks (such as the batteries' energy density growing steadily) can be covered, e.g., Nissan has recently upgraded its model of EV with a range of 250 km, and the cost of the lithium-ion battery shows a 14% reduction per year^4^. There is a need for charging stations at various places along the roads for the charging infrastructure. There is a drawback to transforming the parking lots into vehicle-charging parking lots at various places like hospitals, universities, and railway stations. These vehicles charging parking lots will be powered by wind and solar power, which are not dependent on the grid. For a country, this can provide a great economic benefit. Many countries, such as the United Kingdom, Germany, and Denmark, are working on this thing. As explained above, the described charging structure is not dependent on the grid, but it remains attached to the grid for a two-way flow of power through the usage of grid-to-vehicle and vehicle-to-grid technologies (V2G) in emergencies^5^. Due to high economic growth and population growth, most of the energy consumption growth is in the transportation sector. Excessive emission of carbon dioxide and an energy crisis cause a rapid increase in energy demand^6^. In various countries, mitigation plans have been made to achieve a reduced emission target, and electrifying transportation is also a promising solution. As EVs emit no exhaust gases and produce minimal noise, they can be considered as a substitute for transportation options. EVs have greater efficiency and less operating cost in contrast to the ordinary internal combustion engine vehicle. Fast charging technology and the continuous development of lithium-ion batteries are the major facilitators for the rollout of EVs^7^. Many technical drawbacks, e.g., high initial price, fewer facilities for charging, less range of driving, and long period required by the battery to recharge, are being encountered by the present EV industry.

**Figure 1:**
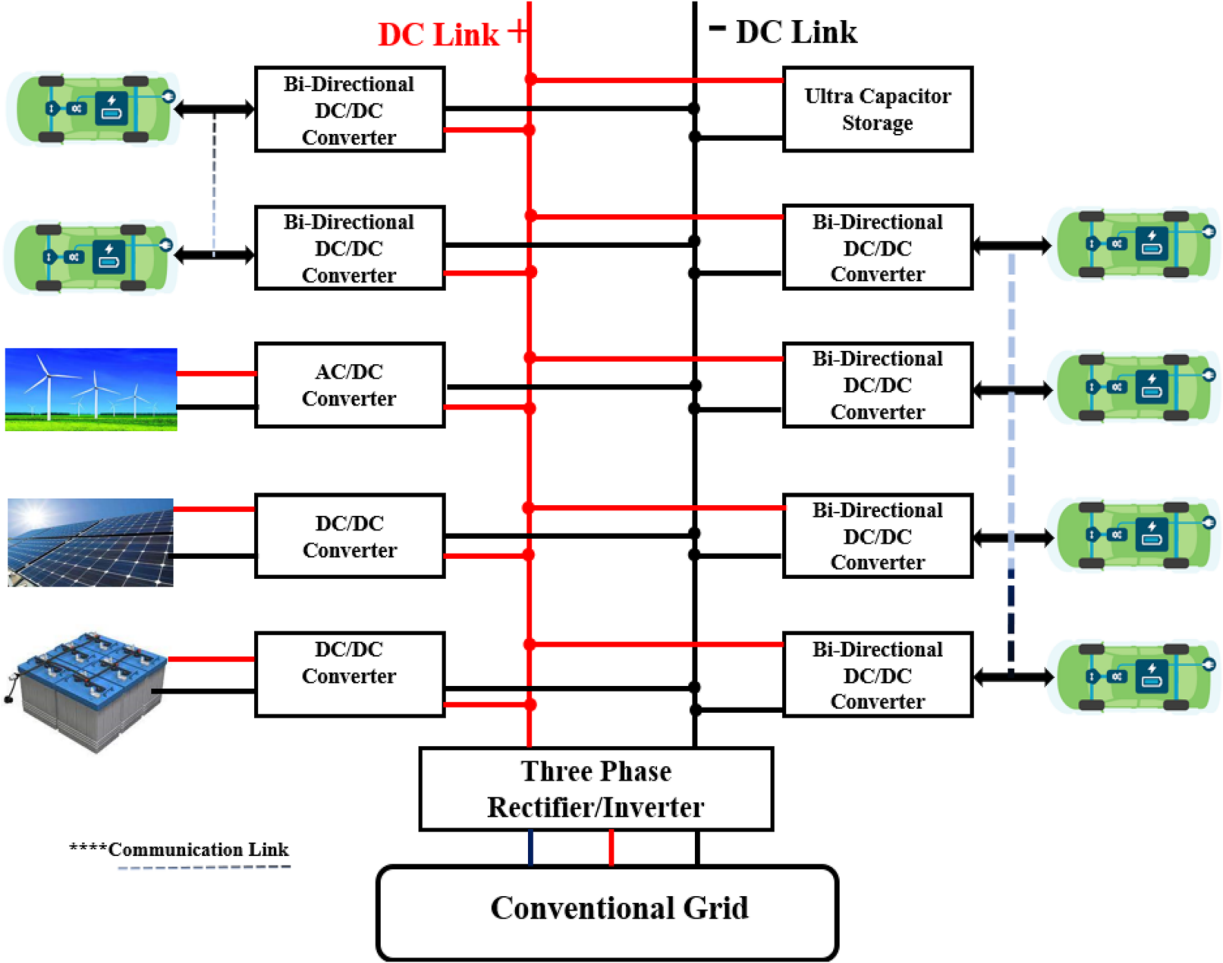
Presence of Bi-Directional DC/DC Converter in Power Grid Setup.

Moreover, adverse effects on the power grid station have been introduced due to V2G technology to receive the charging current. Currently, the power system is modernized with additional communications due to the latest advancements in the smart grid. The V2G concept is a smart grid application that involves electric vehicles to make power systems operate better. It permits the transfer of energy between the electric vehicle and the power grid^8^. Currently, the EV market is witnessing a complete bunch of new producers, e.g., Google, Chevrolet, Ford, and Tesla. The power reduces the sink size and maximizes the transfer of power. The usage of these fewer power losses decreases the switching losses as well as the conduction losses. Ultimately, the power density of the converters has increased^9^. In this regard, the vehicle’s technical performance, such as ripples in voltage and current, is associated with the switching frequency of the converter. Lower voltage ripples are produced by higher switching frequencies in fast-charging units. In the magnetic cores, the low converter ripple in current decreases the harmonic power loss, heat sink size, and weight as well. Higher switching frequencies permit the usage of the pulse current rating of the switch, which is increased by a pulse width decrease and a frequency increment. More switching losses have a negative impact on the functioning of the DC/DC converter on a higher operating frequency. Currently, the focus is on the optimal switching frequency and efficient converter to get a smoother current, good power density, and voltages with less ripple. With fewer ripples in current and voltage, modern EVs have improved power density, whereas modern converter switches use a higher switching frequency^10^. The most important thing that decreases the switching losses for a similar frequency or a higher frequency is an appropriate choice for the material of the transistor. In grid setup, there is a need to deploy the Bi-Directional DC/DC Converter, which is responsible for giving and taking power from the batteries of the EVs, as shown in Fig. 1.

Utilization of wide band gap (WBD) semiconductors, e.g., GaN and SiC models, paves the way for manufacturers to design for less switching losses at high frequencies^11^. The designers have the option (due to the high switching characteristics) to make a trade-off either by the usage of a similar switching frequency to increase the efficiency or by decreasing the size, price, and weight of the filtering components. By the usage of SiC HEMET or Gallium nitride for computer power supplies, it was found that an efficiency of 99% can be obtained at 100 kHz. The major aspects for gaining his efficiency are a low charge, low on-resistance, and high speed, as explained by Fujitsu semiconductors^12^. For the shipboard of 1 MW motor drive in^13^ and smoother current with low ripple, converter switches should function at a greater frequency to decrease the current and voltage ripples. The important thing is decreasing switching losses for similar frequencies, and higher frequencies are an appropriate choice for the WBD material. Short-channel SiC MOSFETs provide greater saturation velocity with high operating frequency and simultaneously put up with declining characteristics of the device, called short-channel effects, in which the most popular is^14^: (a) decrease in transconductance of the device (gm) (b) Device output conductance(gd) after current saturation onset; (c) change in threshold voltage (VT)of the device to -Ve side; and (d) decrease in breakdown voltage (VBr) of the device. To some extent, Short-channel effects will be managed by the optimization of the device channel and the structure of the buffer layer situated directly below the channel^15–17^. It described that short-channel effects are decreased by making channels easily doped and thin. Song et al.^18^ explained that p-type buffer, which is heavily doped, permits the device to operate at high power and increment in thickness of the layer of the buffer from 2.2 to 6 μm to improve $V_{Br}$ from 38 to 275 V. Nowadays, all are looking for an electric vehicle that has a higher operating temperature and is capable of handling high voltages, high efficiency, high power density, and fast switching.

Fast and efficient charging is a critical aspect of electric vehicles (EVs), where Wide Bandgap (WBG) materials offer promising benefits, such as high switching frequency and low conduction losses. However, these materials are limited in handling high power, which is essential for EV charging. Addressing this problem, we propose a hybrid multimodule DC–DC converter based on a dual-active bridge (DAB) topology. The challenge lies in optimizing fast-charging systems by leveraging the advantages of WBG materials while overcoming their power limitations. This research aims to address this challenge by designing, modeling, and experimentally validating a novel converter that incorporates both IGBTs and MESFETs to improve charging efficiency and power density.

Our research offers a comprehensive contribution to the field of EV charging systems, presenting a novel approach to address the challenges of fast and efficient charging. Key contributions include:

- *Hybrid multimodule DC–DC converter* The design and implementation of a hybrid multimodule DC–DC converter integrating both IGBTs and MESFETs, with the dual-active bridge topology forming the backbone of the system.
- *Improved charging efficiency and power density* The innovative hybrid design leverages the strengths of both IGBTs and MESFETs, enabling us to achieve a remarkable power efficiency of 99.25% and a power density of 10.99 kW/L, a significant improvement compared to existing models.
- *Enhanced control strategies* Our research introduces advanced control strategies to ensure uniform power sharing among modules, contributing to the optimization of charging systems.
- *Experimental validation* We provide experimental validation of our proposed converter using MATLAB simulations, confirming the effectiveness of the hybrid design in delivering fast, efficient charging for EVs.
- *Addressing the power limitations of WBG materials* By overcoming the power limitations of WBG materials, our research expands the applicability of these materials in EV charging systems, opening up new possibilities for faster, more efficient charging solutions.

“Related Work” Section provides a comprehensive review of related studies that have explored wide bandgap (WBG) materials for their application in power converters for electric vehicles. The performance analysis of various semiconductor materials and WBG devices concerning Fault is undertaken within this section. “Wide Bandgap Device Structure and Performance Evaluation” Section of this article covers the research methodology, presenting a small signal model that serves as a foundational tool for the investigation. The results, discussion, and conclusions derived from the research are outlined at the end of the article, offering insights into the effectiveness and potential limitations of the proposed hybrid multimodule DC–DC converter. Tables 1 and 2 are included, defining the symbolic representations of key parameters and providing a list of acronyms, respectively, to facilitate the reader's understanding and interpretation of the study's findings.

**Table 1:** Symbolic representations.

Symbols	Description
$\epsilon$	Electrons mobility
${\upmu }$	Dielectric constant
${\text{ E}}_{{\text{f}}}$	Critical electric field
${\text{V}}_{{\text{g}}} { }$	Gate voltage
${\text{V}}_{{\text{B}}} { }$	Breakdown voltage
${\text{I}}_{{{\text{DS}}}} { }$	Drain to source current
${\text{R}}_{{{\text{DS}}}}$	Drain to source resistance
${\text{I}}_{{\text{S}}}$	Source Current
${\text{m}}_{{\text{f}}}$	Forward transconductance
${\text{gm}}_{{\text{f}}}$	Output transconductance
${\text{V}}_{{{\text{CE}}}}$	Collector-emitter voltages
${\text{I}}_{{\text{o}}}$	Output current
${\text{P}}_{{\text{A}}}$	Power delivered to the first group of modules
$\Delta {\text{D}}_{{{\text{A}}1}}$	Change in duty cycle in the first module
${\text{D}}\Delta {\text{V}}_{{{\text{A}}1}}$	Change in voltage by changing the duty cycle for the first module
${\text{D}}\Delta {\text{I}}_{{{\text{A}}1}}$	Change in current by changing the duty cycle for the first module
${\text{I}}_{{{\text{AT}}}}$	The total current of the first module after a change in duty cycle
${\text{P}}_{{\text{B}}}$	Power was delivered to the second group of modules
$\Delta {\text{D}}_{{{\text{B}}1}}$	Change in duty cycle in the second module
${\text{D}}\Delta {\text{V}}_{{{\text{B}}1}}$	Change in voltage by changing the duty cycle for the second module
${\text{D}}\Delta {\text{I}}_{{{\text{B}}1}}$	Change in current by changing the duty cycle for the second module
${\text{L}}_{{\text{A}}}$	The equivalent inductance of modules of group A
${\text{ C}}_{{\text{A}}}$	Equivalent capacitance of modules of group A
${\text{L}}_{{\text{B}}}$	The equivalent inductance of modules of group B
${\text{ C}}_{{\text{B}}}$	Equivalent capacitance of modules of group B
${\text{R}}_{{{\text{DA}}}}$	Change in the internal resistance due to the duty cycle due to switching frequency in group A
${\text{R}}_{{{\text{DB}}}}$	Change in the internal resistance due to the duty cycle due to switching frequency in group B
${\text{G}}_{{{\text{vDA}}}}$	Transfer function for the output voltage of modules to change in the duty cycle for the second group-A
${\text{G}}_{{{\text{vDB}}}}$	Transfer function for the output voltage of modules to change in the duty cycle for the second group-B
${\text{Z}}_{{\text{A}}}$	The transfer function for the impedance for the modules of Group-A
${\text{Z}}_{{\text{B}}}$	The transfer function for the impedance for the modules of Group-B
${\text{V}}_{{{\text{oA}}}}$	Output voltages of the group-A
${\text{I}}_{{{\text{oA}}}}$	The output current of the group-A
${\text{V}}_{{{\text{oB}}}}$	Output voltages of the group-B
${\text{I}}_{{{\text{oB}}}}$	The output current of the group-B
${\text{PA}}_{{\text{losses }}}$	Power losses by IGBT converters relying on the wide bandgap material
${\text{PB}}_{{\text{losses }}}$	Power losses are caused by the wide bandgap converter relaying on the MESFETs converter
${\uprho }$	Power density

**Table 2:** Acronyms representations.

Symbols	Description
WBG	Wide Band Gap
DAB	Dual-Active Bridge
EVs	Electrical Vehicles
HEV	Hybrid Electrical Vehicles
IPT	Inductive Power Transfer
CPT	Capacitive Power Transfer
V2G	Vehicle-to-Grid
THD	Total Harmonic Distortion
OBC	On Board Charger
DBC	Direct Bonded Copper
OCS	Output Current Sharing
IVS	Input Voltage Sharing
CSI	Current Source Inverter

## Related work

Z. John Shen and his team thoroughly elaborated on the current advancements and development of power semiconductor devices, emphasizing their significance in hybrid, electric, and fuel cell vehicles^19^. Furthermore, with the emergence of wide bandgap (WBG) semiconductor technologies, the usage of WBG-based devices like MOSFETs and IGBTs has gained traction in the electrified transportation sector, as highlighted by the research of Ajay Moray and others^20^. Specifically, WBG devices find critical applications in AC electric drives, particularly in high-speed and low-inductance motors. Moreover, these devices are proving instrumental in achieving operational feasibility in areas characterized by high temperatures, ensuring optimal performance in various scenarios, including high-speed and megawatt-level motors. The inherent capabilities of WBG devices, such as their ability to function at high junction temperatures, have led to their integration into integrated motor devices, further expanding their utility^21^.

EVs can be changed either by contactless approach or by direct contact. Inductive Power Transfer (IPT), a contactless charging approach, has several advantages and disadvantages. Capacitive Power Transfer (CPT) is an alternative method for transferring power wirelessly. Research scholars and scientists discuss Many aspects of CPT^22^. The authors described the quantified study of the usage of fast-switching low-losses Wide band gap devices over traditional Silicon devices in the DC/DC converters’ switching. The fast switching of Silicon Carbide and Gallium Nitride semiconductors decreases switching power losses. A great improvement of 2.2% in switching efficiency is made possible by using the GaN E-HEMT cascade. About 2% improvement in switching efficiency is attained by SiC trench ACCUFET. In all conditions for the tested normal load, the efficiency gap between GaN and SiC switches is constant (about the range of 0.6–0.7%)^23^. In electric vehicles, DC–DC power converters are replaced with DC–DC battery chargers and traction drives. Recent lateral GaN devices are not appropriate for these two power electronic modules. Some researchers have presented the two-phase DC–DC, where one phase is a GaN-based transistor while another phase is a SiC-based MOSFET^24^. A design of a 15,000W DC/DC converter along with LLC topology as SiC devices to decrease the power loss with high efficiency^25^. They showed that their proposed converter was 98.4%, which is 3% higher than the conventional converter. An increment of 27.6% takes place in the power density of proposed converters, whereas volume decreased by 21.9% compared to conventional converters. In this research paper, for level-2 integrated on-board chargers, the contrast of isolation converters based on SiC, Si, and GaN switching devices is presented. Operating constraints and design trade-offs are also explained^26^. In the field of electric vehicles, researchers have reviewed the benefits, framework, and challenges of V2G technology. They summarized major optimization techniques to attain various V2G objectives while satisfying constraints^27^. For low-voltage micro-generators, conventional two-stage power converters, along with bridge rectifiers, are not practicable and are inefficient^28^. They presented an efficient AC/DC power converter that prevents bridge rectification at a higher efficiency, which directly converts low AC input voltage to the desired high DC output voltage. In another research, authors reported that WBG of GaN provides more efficient chargers and converters, which makes electric vehicles efficient and environmentally friendly. They also reported discrete GaN depletion mode HEMT with $V_{Br}$ of 200 V and high isolation resistance of 2.89 × 1010 Ohms/sq and a mobility of 1600 cm^2^/V sec for 48 V DC–DC power converters employed in EVs^29^. The authors presented ways to make an electric vehicle charging system that is highly efficient. The photovoltaic EV charging design is becoming complex due to the various features included in the scheme. Photovoltaic EV charging design requires high-power-density storage, high-performance power switches, quick charging, and dynamic response with increased power quality. SiC and GaN semiconductor switches have fast switching ability due to the less power losses. These switches can operate at high-frequency operation because the size of the converter is decreased^30^.

EV-related trends and global charging standards are summarized in^31^. Different integrated onboard charger (OBC) techniques, i.e., system integration with EVs’ auxiliary power modules and wireless charging systems, are explained. During wireless charging of electric vehicles, ultra-wideband communication is implemented for the exchange of information. The authors in^32^ explained the usage of ultra-wideband devices to control signaling in wireless power transfer for EVs. They also highlighted the potential uses of WBG devices in AC motor drives. To notice the full potential of WBG devices in motor derives, the converter design considerations, technical challenges, and the design trade-off are explained^33^. To deal with the low power rating of GAN, the authors presented two solutions in their research work: two-phase GaN-SiC-based and single-phase paralleled GaN. An instantaneous power loss analysis method is introduced to analyze the contributors of power loss. By implementing the instantaneous method on CAD Spice simulation samples, they demonstrated the paralleled GaN converter’s superiority, i.e., in terms of efficiency^34^. Luca Concari et al. analyzed the performance of a 3-phase converter architecture with decreased common mode voltage that could be implemented in electric motor drives. They worked on three important parameters: efficiency, reliability, and common mode voltage. The reliability analysis was performed using the Coffin-Mason model, which showed that higher efficiency is provided by SiC devices^35^. For future devices, the power conversion topologies that are the most appropriate are explained. Power conversion topologies are current light two-medium HEV/EVs and two-level three-phase topology. Multiphase technologies have been applied in high-power applications. A control scheme is introduced to focus the main attention on the module's practical design aspects, which are the layout of the modules and direct bonded copper (DBC) routing with optimum we band-based die parallelization and placement^36^.

Various scenarios arise due to malicious cyber-attacks. Researchers are working comprehensively and providing recommendations to defend the effect of many data strategies on the power electronic hardware that is in an EV charger. Aritra Ghosh described the challenges faced due to the utilization of EVs in the transport sector for de-carbonization purposes. The main components of EVs are the storage systems and the charging station with efficient power electronics. The EV charging station is mainly given power from the grid station, but it can be replaced with solar energy. The battery of EVs is deficient in tailpipe emissions in contrast to other types of EVs. Therefore, EVs are thought to be true zero-emission vehicles^37^. Yuanjian Zhang et al., in their research work for EVs, designed an optimal control strategy in which IoVs are incorporated. As compared to the original strategy, the simulation result shows the greater performance of the novel optimal control technique for EVs^38^. Currently, there are many new developments aimed at making improvements in EVs and their parts. This plays a pivotal role in developing the next EV generation. Many novel EV technologies are also explained. Potential design, modeling, and potential technological challenges are also addressed in^39^. A research survey regarding various GaN devices-based DC–AC, AC–DC, and DC–DC converters along with their features has been conducted; methods for solving the issues of power modules, i.e., parasitic, thermal design, and layout other than that of the power converters are also provided in^40^. For evaluating the performance of wireless chargers, a figure of merit (FOM) is proposed for the mini scale^41^. Efren Fernandez et al., in contrast to the conventional Si devices, SiC-based switching devices have provided improvements in performance in various aspects, including high operating temperatures, lower power dissipation, and faster switching. SiC devices are used in the CSI topology, which is thought to be emerging. This work describes techniques to decrease total harmonic distortion (THD) output currents of current source inverter (CSI) topology by a V–I power converter based on SiC. In this method, the switching frequency and the phase-shift angle between the two carrier signals are adjusted. The efficiency is enhanced by changing the phase angle between the Pulse width modulation carriers of both the power converter switching modulators, i.e., V–I and CSI. Panbao Wang et al. presented in their research for high-speed SiC MOSFETs, whereas a crosstalk suppression method is proposed. By this method, a crosstalk voltage is decreased by incrementing the gate to source capacitance and decreasing the wide band gap MESFETs' output characteristics. In this regard, a better temperature-dependent analytical model is presented by S. Rehman et al. In this modeled data, improvement is made due to a comparative analysis of modeled and observed characteristics. To assess the Miller capacitance, the analytical expressions are developed for linear and saturation regions of operation^42^. An intelligent control method for DC fast charging stations is introduced in^43^, where a control strategy is presented to control the voltage fluctuations. The rises and drops of voltage are also challenging tasksin electric vehicles as they cause instability in modern power systems^44^. The power systems need a strong network like IoTs to manage the charging schedule of the latest V2G technology^45^. Multilevel converters have been introduced in the latest EV chargers because these converters can handle higher voltage values with lower THD^46,47^. On the other hand, cascade topology is also in debate for battery chargers of EVs as variation in SOC of the batteries is limiting the performance^48,49^. The property of DC–DC converters is that they can regulate the wide range of voltage for the on-boarding charging system^49^. The present study supports the beneficial results associated with multilevel converters in cascade topologies, as previously documented in the literature. Specifically, the suggested paradigm is influenced by a multimodule pattern described in reference^50^. This novel idea seeks to tackle the present obstacles related to ultra-fast electric vehicle (EV) chargers, with a particular focus on the simultaneous achievement of both high power and high switching capabilities^51–53^. The model stands out due to its utilization of a dual-group architecture of multimodule converters, with each converter specifically tailored to meet different operating needs^54^. The initial group is tasked with the duty of overseeing high power management, utilizing Insulated Gate Bipolar Transistors (IGBTs) as they have demonstrated their capacity to handle increased power levels effectively. In contrast, the second category, which focuses on demanding switching needs, incorporates switches made with Metal–Oxide–Semiconductor Field-Effect Transistors (MOSFETs)^55^. The design justification for this is based on the inherent benefits of MOSFETs, namely their superior switching speeds and reduced conduction losses^56^. The second group's use of wide bandgap (WBG) materials becomes crucial in response to the increasing need for ultra-fast charging in electric vehicles (EVs)^57,58^. Materials like silicon carbide (SiC) and gallium nitride (GaN), which are part of the wide bandgap (WBG) family, provide exceptional material features that allow for increased switching frequencies and reduced power consumption^59,60^. The deliberate distribution of converter duties guarantees a well-rounded strategy that combines the power-handling capacities of IGBTs with the switching efficiency of MOSFETs^61,62^. This technique effectively tackles the dual obstacles of high power and high switching. Although combining both groups has the potential to reach extremely high switching frequencies, it is important to recognize the intrinsic constraint of WBG devices—their inability to withstand high power levels^63^. Therefore, it is necessary to use a hybrid strategy that utilizes the advantages of both conventional and wide bandgap (WBG) devices in order to achieve an ideal equilibrium between power and switching needs^64,65^. Expanding on the findings of reference^50^, the study emphasizes the existing lack of WBG devices and highlights the urgent requirement for their incorporation into EV fast-charging applications. In order to strengthen the theoretical basis of the suggested model, a comprehensive small-signal model for the converter is explained^66^. This model is crucial in developing a control approach that aims to achieve both the intended power-sharing dynamics and the overall stability and dependability of the charging system^67^. To summarize, the main benefit of multilevel converters, demonstrated by the suggested model, is their enhanced control capabilities due to a larger number of available switching states^68,69^. The hybrid design, which combines the most advantageous characteristics of conventional and wide bandgap (WBG) devices, has great potential for the development of high-speed electric vehicle (EV) chargers. This configuration effectively tackles the complex issues related to power and switching requirements in a comprehensive way^70^.

The current revolution in WBG materials encourages fast switching where these devices are not capable of tackling high power. However, conventional converters have high power ratings but have a very slow switching speed as compared to WBG-based devices. Besides all these problems, the latest research is being strict on the fast charging of EVs. To compensate for these issues, we are developing a hybrid module multimodule-based converter that has a high-power rating with a high switching speed. The proposed model not only increased the power efficiency but also enhanced the power density with high switching as we used the WBG device MESFET in a multimodule converter. The topology we have adopted in the model is DAB, as it needs more attention, especially in grid-connected vehicles, as shown in Fig. 1.

## Wide bandgap device structure and performance evaluation

**Figure 2:**
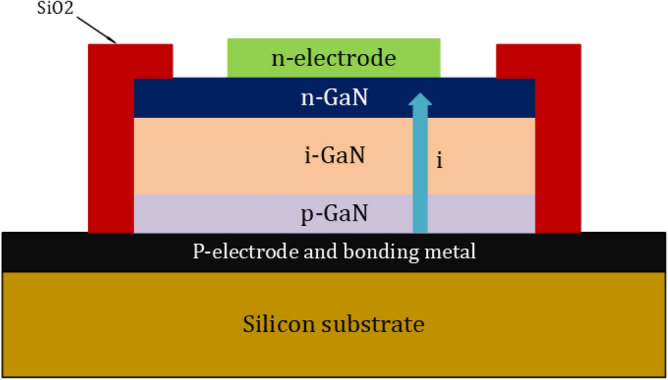
GaN diodes structures. (**a**) Vertical PN^72^.

**Figure 3:**
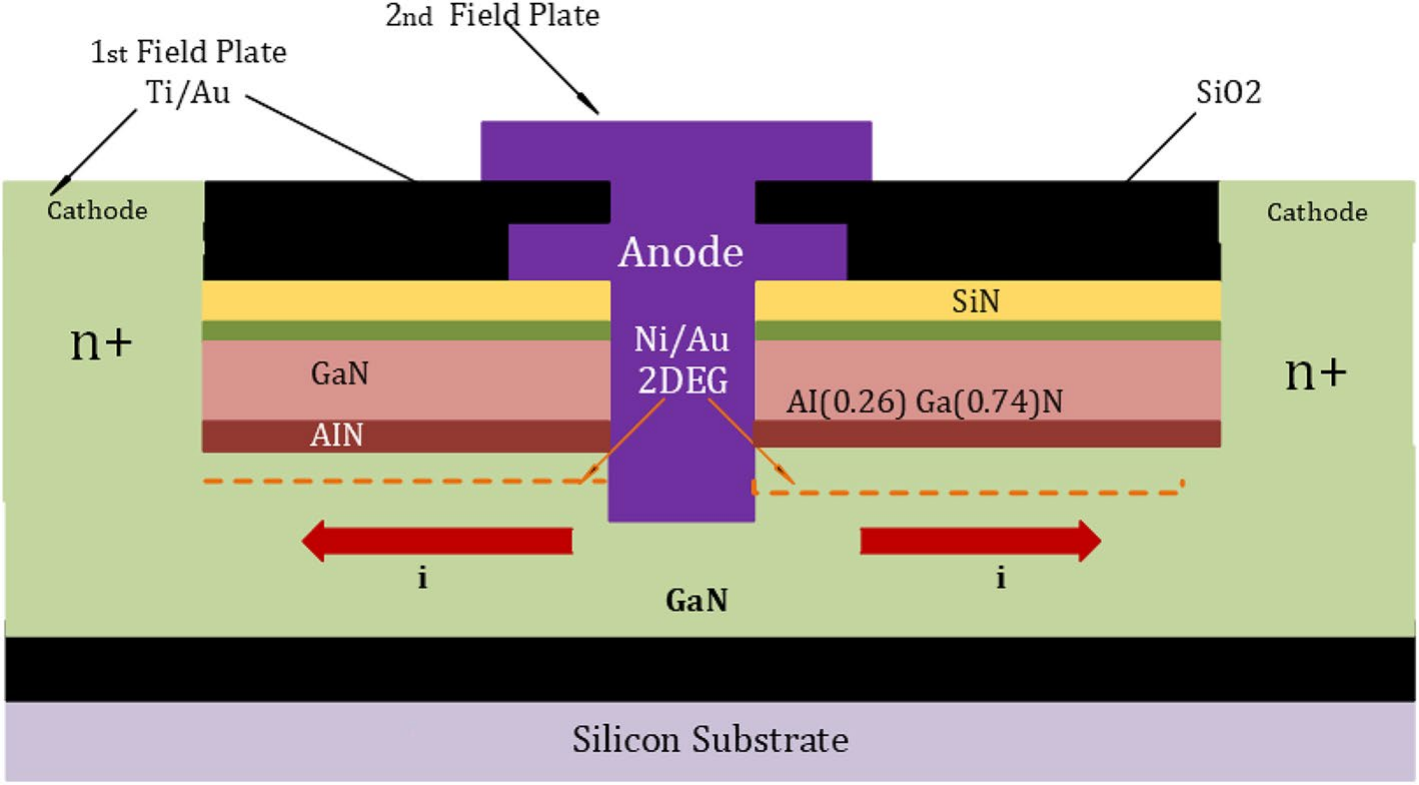
GaN diodes structures. LateralSchottky^73^.

Since SiC bulk wafer fabrication is highly advanced, SiC diodes are suitable for power applications as their manufacturing is based on a vertical structure. The drift area of SiC diodes is substantially thinner than that of Si PN diodes, and this is a reason for the high critical electric field strength of SiC. As a result, substantially less charge is held in the drift region, allowing for fast reverse recovery and high switching speeds. On the other hand, the GaN-on-GaN devices are considered the bulk native GaN substrates that are used to construct GaN power devices. Although the performance of these devices is very enticing, the price of free-standing GaN wafers is too high. Given the cost issues, the development of diodes based on GaN-on-SI wafers has recently been initiated. One of two fundamental configurations has been configured to create GaN-on-Si diodes^71^. The first is the vertical structure, as seen in Fig. 2, while Fig. 3 also shows the GaN-On-Si diodes in their Lateral Schottky configuration. In these latest PN junctions, a 2DEG is constructed at the heterojunction of the AlN and GaN layers. Due to the high electron mobility found in these types of materials, they can achieve high conductivity between the anode and the cathode. It has been observed from the performance analysis of SiC and GaN diodes that the SiC devices can withstand higher temperatures while GaN has the advantages of Low-Threshold Gate Voltage and a Higher rate of voltage changes. Therefore, it has fast switched in both on and off times. Johnson defined the relation for low-rating transistors where the power–frequency product is carried out. After that, the Baliga figure of merit has been introduced. The figure of merits has estimated the impact of material parameters. This modeling of the field distribution is usually done to calculate the intensity of the doping and the width depletion region which is desired to maintain the voltage^72^.

In this regard, the WBG diode performance can be observed by the ideal specific on resistance. It is the resistance per unit area of this layer of material required to support the voltage. This resistance defines the per unit area for the resistance of the specific layer to support the voltage^74^. Hence, the expression is given as:

1 $$R_{{{\text{on}}}} = 4{\text{V}}^{2} /\epsilon \times \mu \times E_{f}^{3}$$

where V is the breakdown voltage, and it indicates the conduction performance of the PN junction. Electron mobility, dielectric constant, and critical electric field denoted by $\epsilon ,{ }\mu ,{\text{ and }}E_{f}$, respectively.

**Figure 4:**
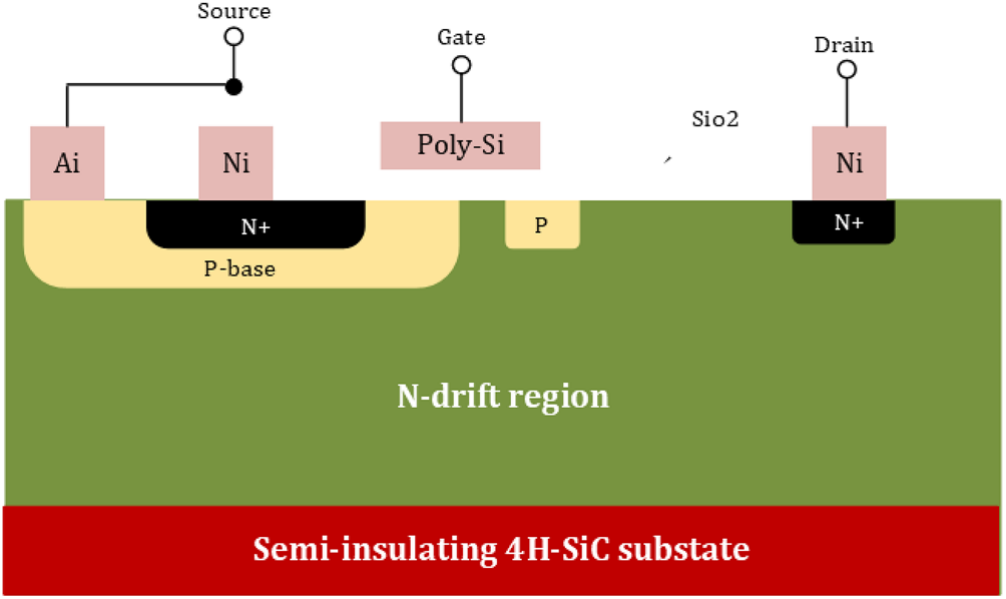
SiC Lateral MOSFETs structures^77^.

**Figure 5:**
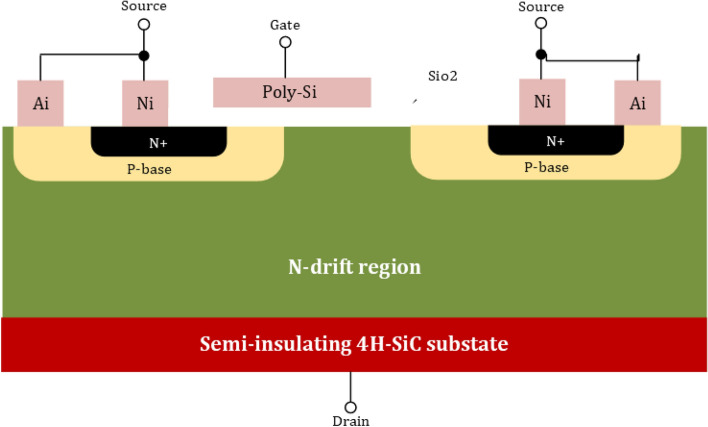
SiC Vertical MOSFETs structures^78^.

**Figure 6:**
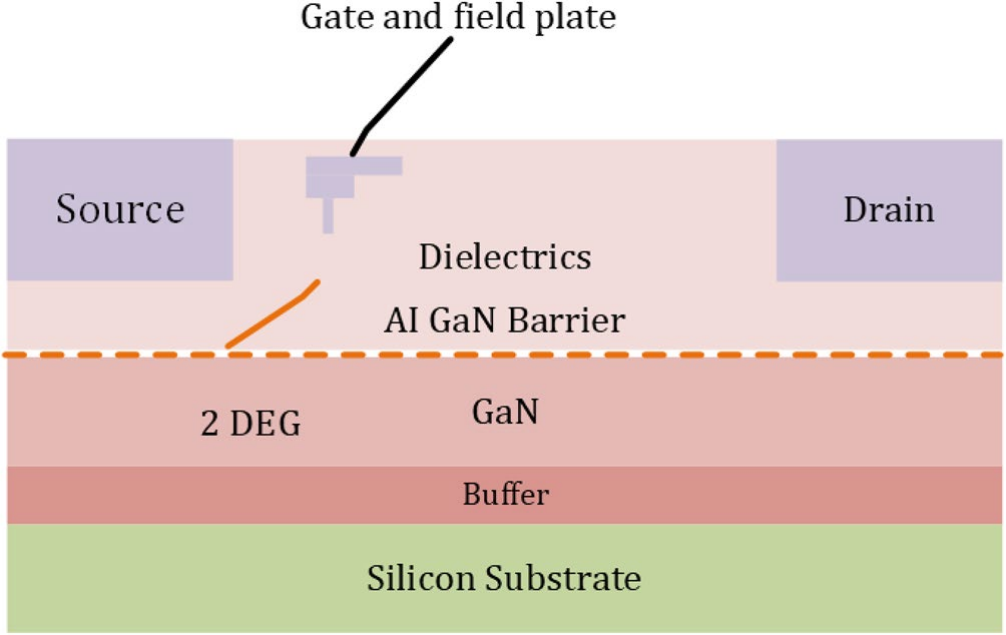
Typical Lateral GaN HEMT Structures^79^.

Now, it comes to the transistor side, where MOSFET and JFETs are the common devices to carry the current only for the majority of careers. SiO2 is commonly considered a stable oxide of SiC to fabricate the SiC MOSFETs^73^. This fabrication is based on the development of the SiC wafer fabrication technology. To analyze the WBG transistor, there is a need for high reverse blocking voltage. The best suitable option to deal with this blocking voltage is SiC Lateral MOSFET structures, as shown in Fig. 4. It is especially well suited for monolithic integration with other circuits since the gate, drain, and source terminals of the device can be adjusted using the top surface. On the other hand, high voltage with high-power applications deals with SiC MOSFETs with a vertical design, as shown in Fig. 5. SiC MOSFET research is moving quite swiftly because the two devices’ structures aren’t all that dissimilar. The performance of the SiC MOSFET with vertical structure is extremely close to its theoretical limit. Affordable SiC MOSFETs are currently widely available. SiC MOSFETs from suppliers like Infineon, Wolfspeed, and ROHM are readily available off the shelf with voltage ratings ranging from 600 to 1700 V. As earlier mentioned in the section, the GaN-On-Si FET has received a lot of research and attention where the reason is the advancement of the wafer production technology. The structure of GaN-On-Si FET is shown in Fig. 6. The discussion is made about the Johnson figure of merit in^75^ and Baliga figure of merit in^76^; the minimum power losses can be written as:

2 $$P_{min} = 2.8\sqrt {\frac{{8I_{{{\text{DS}}}}^{2} \cdot V_{G}^{1.75} \cdot V_{B}^{1.5} \cdot f}}{{\mu \cdot E_{f}^{2} }}}$$

**Figure 7:**
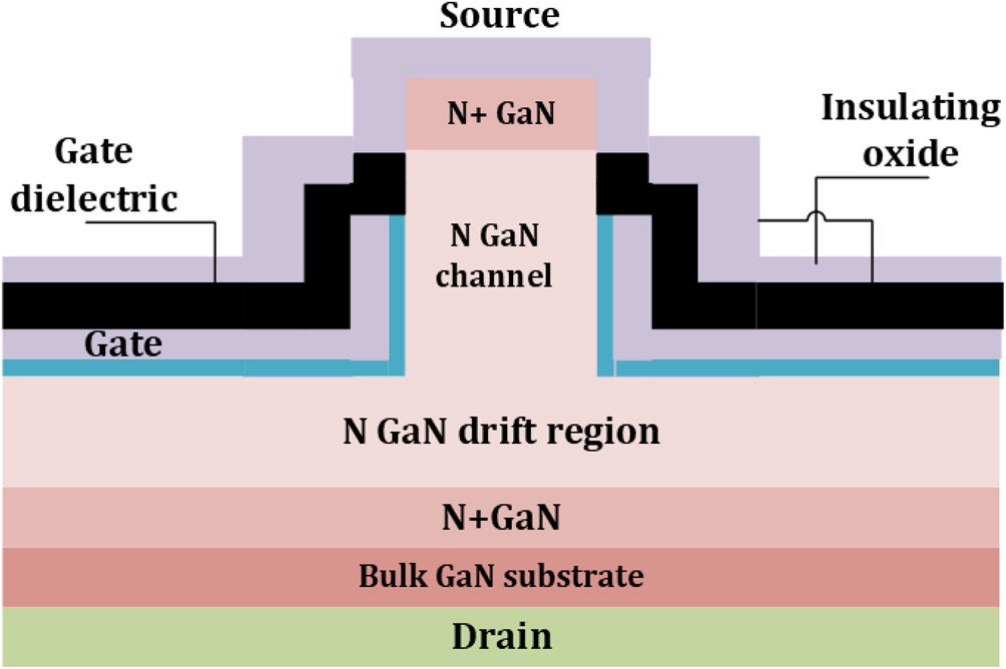
Typical Lateral GaN FinFET Structures^80^.

**Figure 8:**
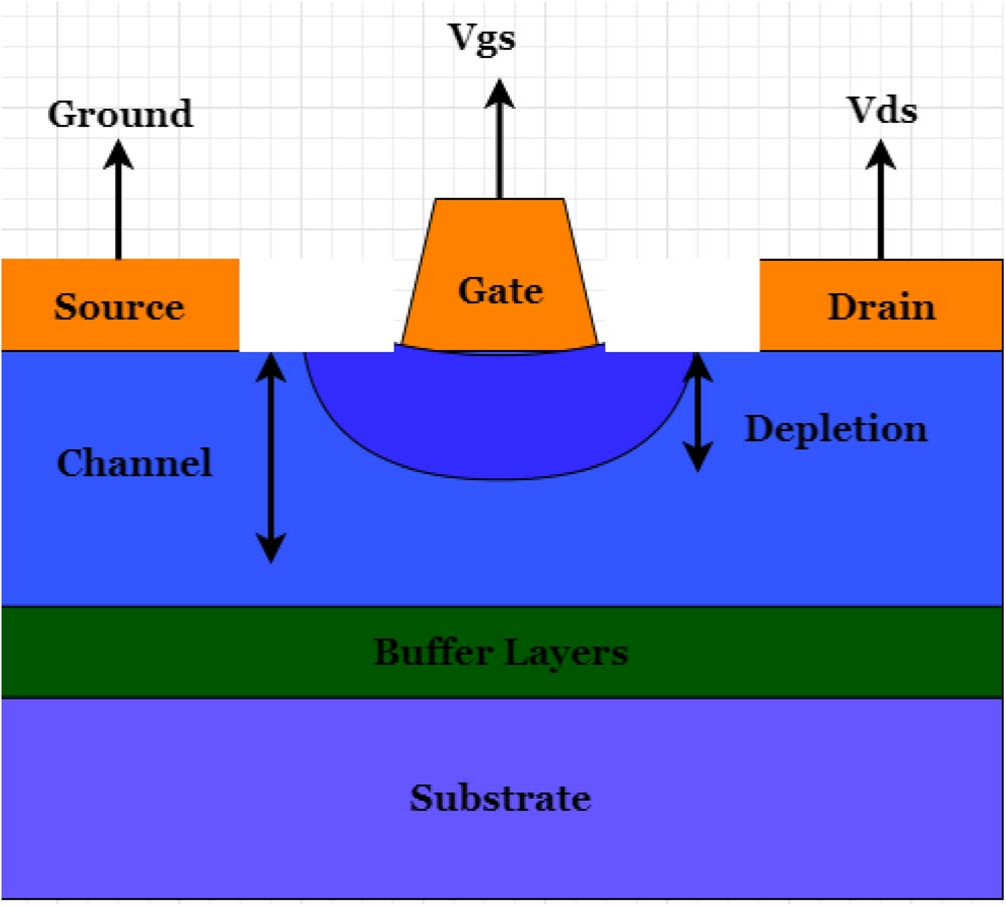
A cross-sectional view of an operating MESFET^42^.

Equation (1) covers only the PN junction, while Eq. (2) deals with Power JFETs. Here $I_{DS}$ is the conduction current of the device, and Vg is the gate voltage. Like the lateral GaN diode, as shown in Fig. 3, the lateral GaN FETs can carry a high electron mobility current using the 2DEGs. This typical device is HEMT, where the gate-to-source bias voltage is zero, and the 2DEG is a normally-on depletion transistor. A cascade low-voltage Si-MOSFET can be applicable for a normally off-power device, but it is not suitable for EVs. A P-GaN layer placed between the gate and the AlGaN barrier can be used to create a genuine normally-off GaN E-HEMT despite the cascade shape, as shown in Fig. 6. The P-GaN layer can be depleted by properly planning the doping concentration and layer thickness^79^. As lateral GaN HEMT is not a junction structure, it has no avalanche effect for lateral GaN WBD. A high level of breakdown voltage can be problematic for a GaN device. In normal conditions, the GaN devices can deal with higher voltages, up to 650–1300 V. In EVs, there is a need to switch transistors with a high dielectric lifespan to create a high breakdown voltage in a substantial part. In this regard, GaN has been recognized as one of the potential materials and recently introduced a new structure of a normally-off vertical GaN-on-GaN Fin FET. This type of transistor based on GaN-on-si wafer is carried out where several advantages have been found as this type has series resistance provided by buffer layer as shown in Fig. 7. Like other FETs, a cross-sectional view of an operating MESFET, illustrating recessed gate technology, is illustrated in Fig. 8. Although power losses of FinFET are also dependent on the resistance $R_{DS}$ same as in Eq. (1) for the PN junction. Here, this can be calculated:

3 $$P_{{losses{ }}} = 4\left( {I_{D} } \right)^{2} \times R_{DS}$$

On the other hand, the power losses in HEMT can be calculated by Eq. (4) as given:

4 $$P_{{losses{ }}} = R_{dyn} \times I_{D}^{2} \cdot D$$

The losses remain dependent on the same parameter as Eqs. (3) and (4) as the $= {\text{V}}_{{{\text{DS}}}} /I_{D}$, but the duty cycle can change its effects on it. When the duty cycle is changed, the V–I characteristics are also changed, and ultimately, the resistance changes the losses as well. The D in the above equation is used for the duty cycle. So there is a need for a function that can include time as well to deal with the duty cycle, then the equation becomes:

5 $$P_{losses } = \frac{1}{T}\int_{0}^{T} {I_{D} \times V_{DS} \cdot dt }$$

where the relation of $I_{D} {\text{ and }}V_{DS}$ can be calculated as:

6 $$I_{D} = gm_{f} \cdot {\text{log}}\left[ {1 + {\text{exp}}\left( {\frac{{V_{GS} - b}}{c}} \right)} \right] \times \frac{{\left( {m + n \cdot V_{GS} } \right)V_{DS} }}{{1 + gm_{o} \cdot \left( {d + e \cdot V_{CS} } \right)V_{DS} }},{ }V_{DS} \ge 0$$

$gm_{f}$ and $gm_{f}$ are used for the forward transconductance and output transconductance, respectively. $V_{GS} { }$ is the gate to source voltage where m, n, d, e are the parameters used for output characteristics to solve the nonlinear equation as given in^41^. In IGBTs, the calculation for the power losses becomes:

7 $$P_{{losses{. }}} = V_{{CE\left( {\text{ max }} \right)}} I_{{o{ }}} \times D$$

Same as FETs, the $V_{DS}$ replaced by $V_{CE}$, the collector-emitter voltages when IGBT is in the saturation region, whereas $I_{o}$ is the output current. Although the FET devices are normally considered for RF application here, we have required the high-power application as an electric vehicle. RF performance of FETs relies mainly upon the device's intrinsic parameters. Parameter extraction techniques play a crucial role in determining the accuracy of the predicted intrinsic parameters. For accurate small-signal modeling, a suitable capacitor model is needed. Such a model could help the design engineer to assess the reliability of the device under changing conditions. This is shown by making an exhaustive review of relevant literature based on the internal parameters that SiC/GaN-based devices can operate at higher drain-to-source voltages and can reasonably mitigate self-heating effects compared to second-generation devices such as GaAs. Self-heating effects are directly proportional to the power handled by the device. Under high bias, the characteristics of the device could degrade, and a simple model, either analytical or numerical, may not be accurate enough to predict the device's performance. So, to get a better understanding and wide applicability, there is a need to develop a model for SiC/GaN MESFETs that incorporates self-heating effects.

Moreover, a detailed overview of FETs (MESFETs and HEMTs) is given in this section. Device performance with different aspects is reported. The performance of a FET depends upon the material used for its fabrication. It has been observed that devices fabricated using SiC and GaN have superior performance, both in DC and RF domains, compared to GaAs MESFETs. SiC MESFETs have excellent heat conduction in high-power applications and show maximum stability in performance^42^. By using work done by different researchers, it is shown that SiC MESFETs have great potential to be used in harsh environments. In this regard, multimodule converters have recently been introduced and are expected to be a viable option for the UFC charger as these provide high voltage and high power^81–84^. Low-power modules are the reason for increasing system complexity, overall cost, and conduction losses. On the other hand, the lower number of power modules can also raise the issue of limiting the switching frequency, less power density, and increasing weight and size as well. In the next section, we introduce the multimodule converters that have capabilities to handle high frequency, high-power, high-power density, low conduction losses, and fast charging for vehicles^84–88^.

After performance analysis, choosing a Wide Bandgap Device for Electric Vehicle systems is a hot talk in today’s fast-switching converters. The power converters should be highly efficient and provide a high-power density. Ultimately, they reduce the size and weight as well. By developing a model with only conventional converters (IGBTs), we cannot meet the latest trend of high switching, as Table 3 shows. The WBG materials have the ability to switch fast, have high power efficiency and density, and have low switching losses. These materials can take a limited power fraction from the grid; that’s why a Hybrid topology is introduced in the next section.

**Table 3:** Performance Analysis of WBG-based Devices.

Parameters	IGBTs	MESFETs	FinFETs
Threshold voltage VT	Very Low	Low	High
Trans conductance GM	Very Low	Low	High
Breakdown voltages	Very Low	Low	High
Capacitance	Less varies	Monotonically Varies	Decrease rapidly
Operating voltages	Low	High	Very high
Power handling capacity with fast switching	Very Low	High due to high operating voltages	Very high
Power density	Low	High due to high operating voltages	Very high
Power added efficiency	Low	High	Very high
Operating temperature	Less than 400 C	500 Celsius	700 Celsius
Drain to source resistance RDS	–	High	low

## Research methodology

**Figure 9:**
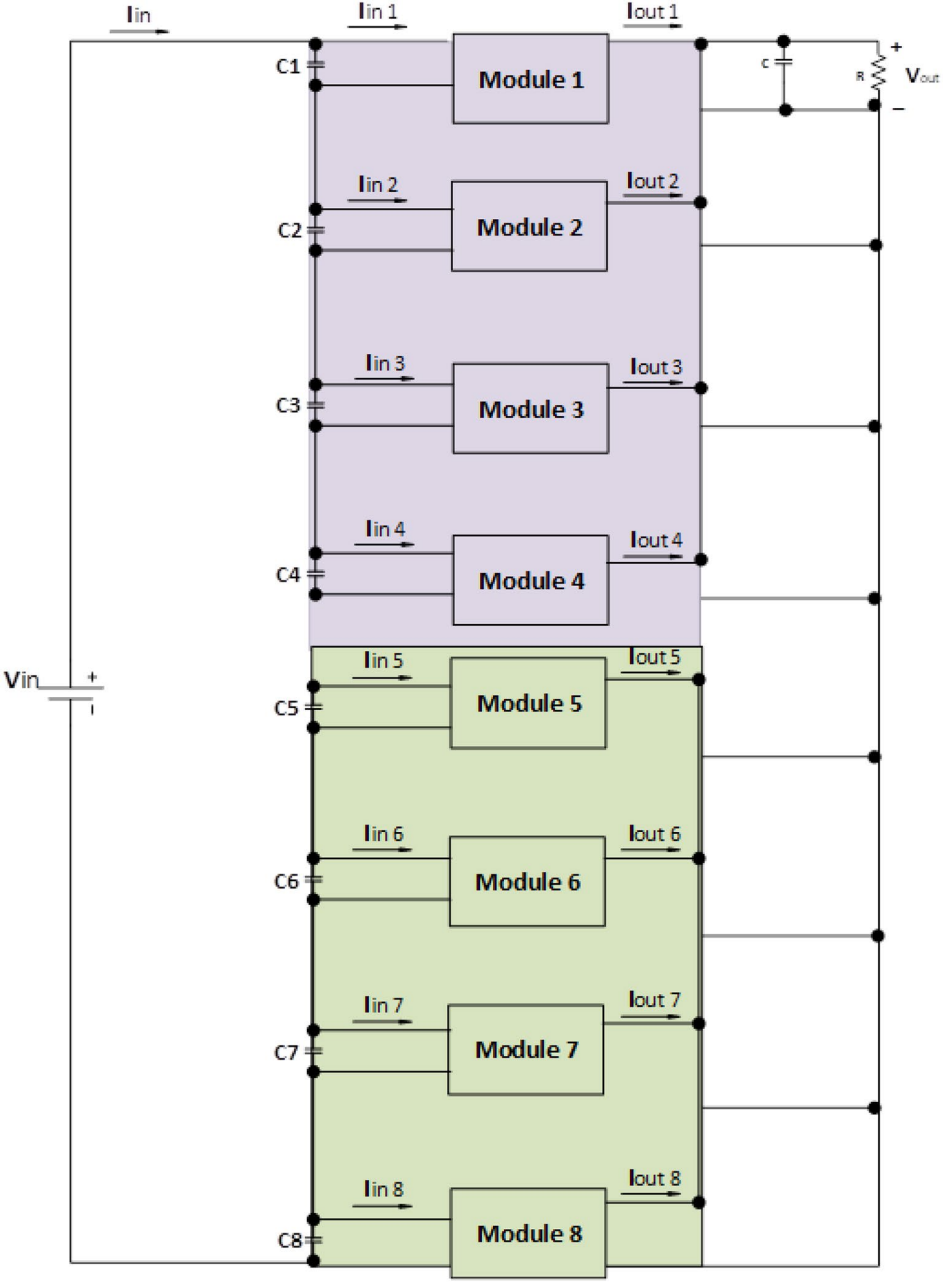
Proposed Hybrid Multimodule Converter Configuration.

In the previous section, we have chosen a MESFET for the Converters, and it is also decided that there is a need for a multimodule converter for the above to obtain the above-mentioned advantages. Some of the research is being conducted on the multimodule pattern, but a concept provided by^50^ is based on two different groups of multimodule converters. The concept is that the first group has a low switching frequency with a very high fraction of the total power. The second group is based on wide bandgap material which has a high switching frequency with a lower fraction of power. Both groups of wideband devices can provide ultra-high switching frequency, but these are not able to handle high power as the charging of the vehicle systems is required. In^50^, the first group is based on IGBTs, and the Second group is based on switches of MOSFETs where the wideband gap was not introduced. We have proposed the same type of hybrid group, but the first one is based on IGBTs (for high power fraction), and the second group consists of a wide bandgap device, MESFET (for high switching frequency), as shown in Fig. 9. The work presented in this paper includes a generalized small-signal model for the presented converter as well as the control strategy required to achieve uniform power-sharing between the employed modules. Besides, a power loss evaluation has been conducted to compare the proposed converter with the other two options. To verify the presented concept, the number of modules needed to achieve the required ratings is calculated for both conventional multimodule DC–DC converters and hybrid multimodule DC–DC converters. In addition, the power loss analysis of the hybrid multimodule converter is provided.

**Figure 10:**
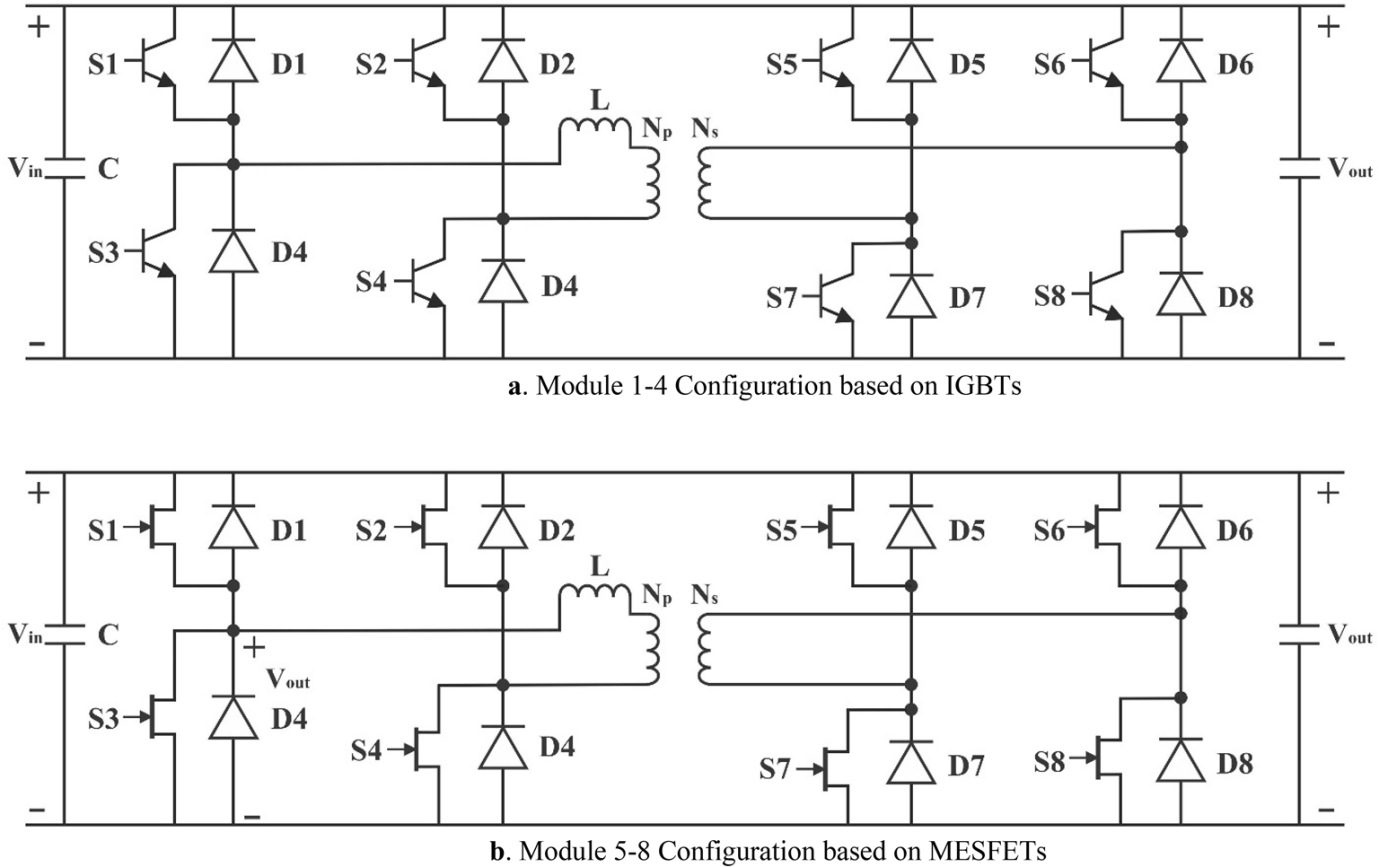
(**a**) Module 1–4 Configuration based on IGBTs. (**b**) Module 5–8 Configuration based on MESFETs.

We have adopted a DAB topology where Module-1 to Module-4 are identical, and IGBTs are used for switching as high-power fractions, as shown in Fig. 10a^89–91^. On the other hand, Modules 5–8 are also identical, but these have used the wideband gap device MESFET to deal with high switching, as shown in Fig. 10b.

**Figure 11:**
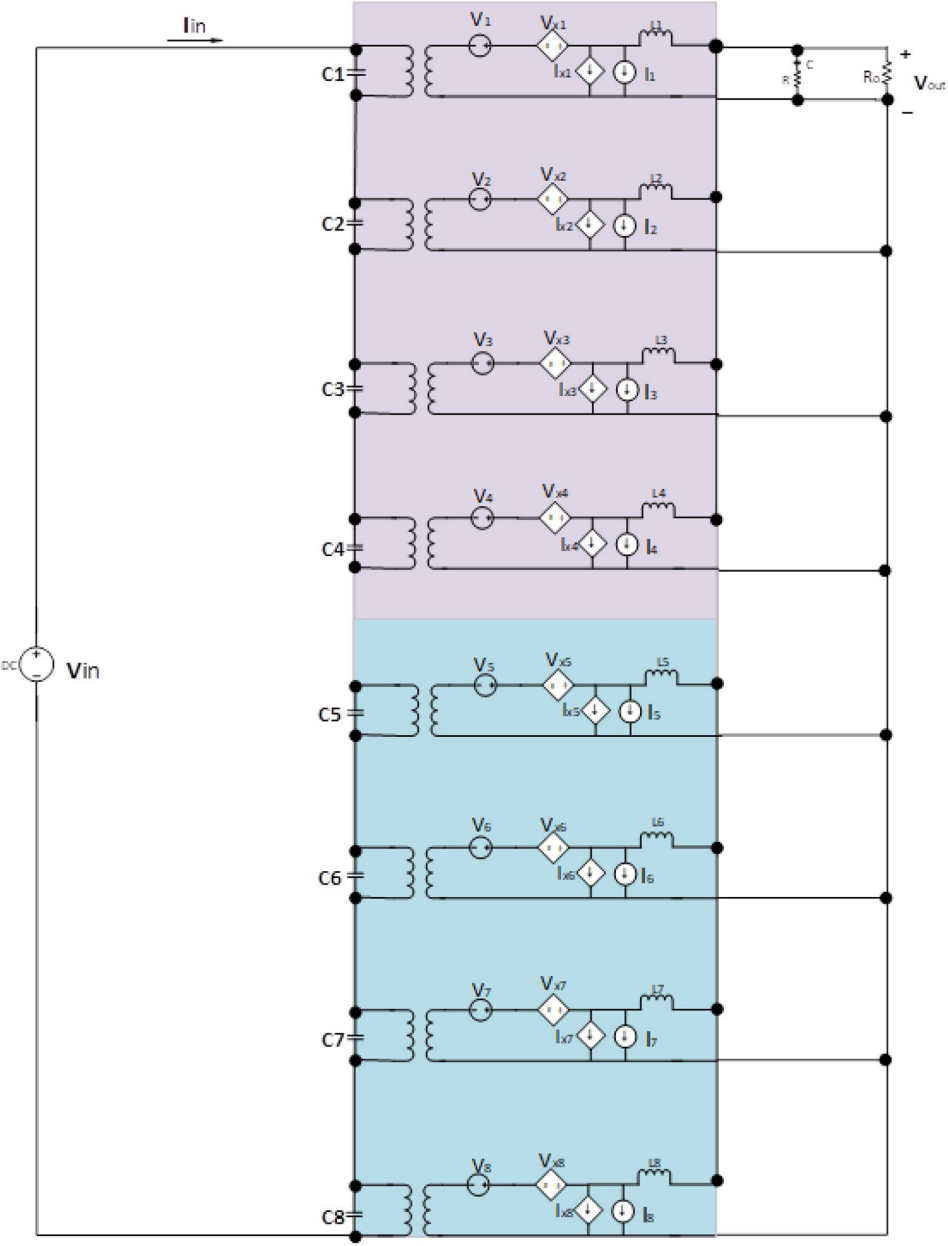
Small Signal Model Configuration of Proposed Hybrid Multimodule Converter.

So, the first group is responsible for dealing with high power and low frequency, whereas the second group caters to low power with high frequency. We are assuming that in our proposed model, the power to be delivered to the 440 V battery is 500 kW. As per Fig. 1, the voltage of the grid is assumed to be 11 kV. In this regard, the first group deals with a high portion of the power, that is, 85% of the total power, 500 kW, which becomes 425 kW. Similarly, the second group deals with the lower portion of power, which is 15% of total power, which becomes 75 kW. The switching frequencies are proposed to be 10 kHz and 200 kHz for the first and second groups, respectively. So, the voltage injected from the grid to the first group is 17/20 of 11 kV, which is 9.35 kV, and the second group takes a voltage of 3/20 of 11 kV, which is 1.65 kV. The charging current required for a battery is 600 A; the first group is responsible for 17/20 of 600 A, which is 510 A, and the second group can provide 3/20 of 600 A, which is 90A. The fractions 17/20 and 3/20 are denoted by K1 and K2, respectively. As there are different current and voltage ratings, there is a need for equal input voltage sharing (IVS) and equal output current sharing (OCS). Hence, the voltages of the first module have been reduced to $V_{inA} /17,$ and the current has reduced to $I_{inA} /17$. The terms $V_{inA}$, $V_{inB}$, $I_{inA}$, $I_{inB}$ are used for the voltages and currents of the first and second groups, respectively. These specifications are modeled in a small signal model, as shown in Fig. 11.

The small signal is presented in Table 4 defines the formulation of the small signal analysis of the hybrid 8-module converter^92,93^. The design is interlinked with “Related Work” Section, where the wideband gap materials’ performances are evaluated, where it is formulated that there would be a change of internal resistance after changing the switching frequency. As we are proposing a hybrid multimodule converter that can affect the input voltage and current by changing the duty cycle to provide a uniform charging current to the batteries of the vehicles. The issues raised are that these changings can affect the inductance and capacitance of the materials^94,95^.

**Table 4:** Small signal formulation of the proposed hybrid multimodule converter.

Item	Formulation	Item	Formulation	Item	Formulation	Item	Formulation
First Group Modules							
$V_{1}$ =	$17\frac{{v_{in} }}{{20P_{A} }}\left( {\Delta D_{A1} } \right)$ Where $P_{A}$ is the power delivered to the first group of modules, and $\Delta D_{A1}$ is a change of duty cycle by fraction 17/20 in the first module	$Vx_{1}$ =	$17\frac{{v_{in} }}{{20P_{A} }}\left( {D\Delta I_{A1} + D\Delta V_{A1} } \right)$ ${\text{Where }}D\Delta V_{A1}$ and $D\Delta I_{A1}$ are the change in voltage and current by changing the duty cycle for the first module in the first group	$Ix_{1}$ =	$I_{AT} \left( {D\Delta I_{A1} + D\Delta V_{A1} } \right)$ Where $I_{AT}$ is the Total current of the first module after a change in duty cycle	$I_{1} { }$ =	$I_{AT} \left( {\Delta D_{A1} } \right)$
$V_{2}$ =	$17\frac{{v_{in} }}{{20P_{A} }}\left( {\Delta D_{A2} } \right)$ ${\text{Wher }}\Delta D_{A2}$ is a change of duty cycle by fraction 17/20 in the second module	$Vx_{2}$ =	$1\frac{{v_{in} }}{{20P_{A} }}\left( {D\Delta I_{A2} + D\Delta V_{A2} } \right)$ ${\text{Where }}D\Delta V_{A2}$ and $D\Delta I_{A2}$ are the change in voltage and current by changing the duty cycle for the second module in the first group	$Ix_{2}$ =	$I_{AT} \left( {D\Delta I_{A2} + D\Delta V_{A2} } \right)$	$I_{2} { }$ =	$I_{AT} \left( {\Delta D_{A2} } \right)$
$V_{3}$ =	$17\frac{{v_{in} }}{{20P_{A} }}\left( {\Delta D_{A3} } \right)$ ${\text{Wher }}\Delta D_{A3}$ is a change of duty cycle by fraction 17/20 in the third module	$Vx_{3}$ =	$17\frac{{v_{in} }}{{20P_{A} }}\left( {D\Delta I_{A3} + D\Delta V_{A3} } \right)$ ${\text{Where }}D\Delta V_{A3}$ and $D\Delta I_{A3}$ are the change in voltage and current by changing the duty cycle for the third module in the first group	$Ix_{3}$ =	$I_{AT} \left( {D\Delta I_{A3} + D\Delta V_{A3} } \right)$	$I_{3} { }$ =	$I_{AT} \left( {\Delta D_{A3} } \right)$
$V_{4}$ =	$17\frac{{v_{in} }}{{20P_{A} }}\left( {\Delta D_{A4} } \right)$ ${\text{Where }}\Delta D_{A4}$ is a change of duty cycle by fraction 17/20 in the fourth module	$Vx_{4}$ =	$17\frac{{v_{in} }}{{20P_{A} }}\left( {D\Delta I_{4} + D\Delta V_{A4} } \right)$ ${\text{Where }}D\Delta V_{A4}$ and $D\Delta I_{A4}$ are the change in voltage and current by changing the duty cycle for the fourth module in the first group	$Ix_{4}$ =	$I_{AT} \left( {D\Delta I_{A4} + D\Delta V_{A4} } \right)$	$I_{4} { }$ =	$I_{AT} \left( {\Delta D_{A4} } \right)$
Second group modules							
$V_{5}$=	$\frac{{v_{in} }}{{85P_{B} }}\left( {\Delta D_{B5} } \right)$ Where $P_{B}$ is the power delivered to the second group of modules, and $\Delta D_{B5} { }$ is a change of duty cycle by fraction 1/85 in the fifth module	$Vx_{5}$=	$\frac{{v_{in} }}{{85P_{A} }}\left( {D\Delta I_{B5} + D\Delta V_{B5} } \right)$ ${\text{ Where }}D\Delta V_{B5}$ and $D\Delta I_{B5}$ are the change in voltage and current by changing the duty cycle for the fifth module in the second group	$Ix_{5}$=	$I_{BT} \left( {D\Delta I_{B5} + D\Delta V_{B5} } \right)$ Where $I_{BT}$ is the Total current of the second module after a change in duty cycle	$I_{5} { }$=	$I_{AT} \left( {\Delta D_{B5} } \right)$
$V_{6}$=	$\frac{{v_{in} }}{{85P_{B} }}\left( {\Delta D_{B6} } \right)$ ${\text{Where }}\Delta D_{B6}$ is a change of duty cycle by fraction 1/85 in the sixth module	$Vx_{6}$=	$\frac{{v_{in} }}{{85P_{A} }}\left( {D\Delta I_{B6} + D\Delta V_{B6} } \right)$ ${\text{ Where }}D\Delta V_{B6}$ and $D\Delta I_{B6}$ are the change in voltage and current by changing the duty cycle for the sixth module in the second group	$Ix_{6}$=	$I_{BT} \left( {D\Delta I_{B6} + D\Delta V_{B6} } \right)$	$I_{6} { }$=	$I_{AT} \left( {\Delta D_{B6} } \right)$
$V_{7}$=	$\frac{{v_{in} }}{{85P_{B} }}\left( {\Delta D_{B7} } \right)$ ${\text{Where }}\Delta D_{B7}$ is a change of duty cycle by fraction 1/85 in the seventh module	$Vx_{7}$=	$\frac{{v_{in} }}{{85P_{A} }}\left( {D\Delta I_{B7} + D\Delta V_{B7} } \right)$ ${\text{ Where }}D\Delta V_{B7}$ and $D\Delta I_{B7}$ are the change in voltage and current by changing the duty cycle for the seventh module in the second group	$Ix_{7}$=	$I_{BT} \left( {D\Delta I_{B7} + D\Delta V_{B7} } \right)$	$I_{7} { }$=	$I_{AT} \left( {\Delta D_{B7} } \right)$
$V_{8}$=	$\frac{{v_{in} }}{{85P_{B} }}\left( {\Delta D_{B8} } \right)$ ${\text{Where }}\Delta D_{B8}$ is a change of duty cycle by fraction 1/85 in the eighth module	$Vx_{8}$=	$\frac{{v_{in} }}{{85P_{A} }}\left( {D\Delta I_{B8} + D\Delta V_{B8} } \right)$ $8{\text{ arehere }}D\Delta V_{B8}$ and $D\Delta I_{B8}$ are the change in voltage and current by changing the duty cycle for the eighth module in the second group	$Ix_{8}$=	$I_{BT} \left( {D\Delta I_{B8} + D\Delta V_{B8} } \right)$	$I_{8} { }$=	$I_{AT} \left( {\Delta D_{B8} } \right)$

To keep the uniform charging current, we formulated the transfer function for different terms^50^. First, the relation of the duty cycle with the output voltage for the module of the first group has been formulated. The transfer function for the output voltage of modules changes in the duty cycle for the second group, A.

8 $$G_{vDA} = \frac{{v_{out} }}{\Delta DA} = \frac{{17\frac{{V_{in} }}{{20P_{A} }}}}{{s^{2} L_{A} C_{A} + s\left( {\frac{{L_{A} }}{R} + R_{DA} C_{A} } \right) + \frac{{R_{DA} }}{R} + 4}}$$

where $L_{A} {\text{ and }}C_{A}$ are the equivalent inductance and capacitance of modules of group-A. $R_{DA}$ is a change in the internal resistance due to the duty cycle due to switching frequency. Similarly, the transfer function for the output voltage of modules changed in the duty cycle for the second group, B.

9 $$G_{vDB} = \frac{{v_{out} }}{\Delta DB} = \frac{{\frac{{V_{{\text{in }}} }}{{20P_{B} }}}}{{s^{2} L_{B} C_{B} + s\left( {\frac{{{\text{K}}L_{B} }}{{\text{R}}} + R_{DB} C_{B} } \right) + \frac{{R_{DB} }}{R} + 4}}$$

where $L_{B} {\text{ and }}C_{B}$ are the equivalent inductance and capacitance of modules of the group B $R_{DB}$ is the change in the internal resistance due to the duty cycle and switching frequency. K defines the fraction that reduces the power for the modules of Group-B. So, the transfer function for the impedance for the modules of Group-A and Group-B can be calculated in (10) and (11).

10 $$Z_{A} = \frac{{V_{oA} }}{{I_{oA} }} = \frac{{\left( {R_{DA} + sL_{A} } \right)}}{{s^{2} L_{A} C_{A} + s\left( {\frac{{L_{A} }}{R} + R_{DA} C_{A} } \right) + \frac{{R_{DA} }}{R} + 4}}$$

11 $$Z_{B} = \frac{{V_{oB} }}{{I_{oB} }} = \frac{{\left( {R_{DB} + sL_{B} } \right)}}{{s^{2} L_{B} C_{B} + s\left( {\frac{{L_{B} }}{R} + R_{DB} C_{B} } \right) + \frac{{R_{DB} }}{R} + 4}}$$

where $V_{oA}$ and $I_{oA}$ are output voltages and current of the group-A. Similarly, $V_{oB}$ and $I_{oB}$ are output voltages and currents of the group-B.

12 $$Z_{A} = \frac{{\frac{{D_{{\text{f1 }}} }}{PA}\left( {1 + \frac{{R_{DA} }}{5R}} \right)}}{{s^{2} L_{A} C_{A} + s\left( {\frac{{L_{A} }}{R} + R_{DA} C_{A} } \right) + \frac{{R_{DA} }}{R} + 4}}$$

13 $$Z_{B} = \frac{{\frac{{D_{{{\text{f }}2}} }}{{P_{B} }}\left( {1 + \frac{{R_{DB} }}{20R}} \right)}}{{s^{2} L_{B} C_{B} + s\left( {\frac{{L_{B} }}{R} + R_{DB} C_{B} } \right) + \frac{{R_{DB} }}{R} + 4}}$$

$D_{{\text{f1 }}}$ and $D_{{\text{f2 }}}$ are the difference in power rating from actual power for Group-A and Group-B respectively.

To evaluate the efficiency of our proposed hybrid modules converter, we must calculate the power losses in both Groups of modules as per the ratings of Table 5^96–98^. In “Wide Bandgap Device Structure and Performance Evaluation” Section, the power losses of IGBTs and MESFETs are estimated in Eqs. (5) and (7). Here, we have four modules in each group. Therefore, the multiplying factor 4 is multiplied, and cumulative power losses in both groups of modules are calculated as:

14 $$PA_{{\text{losses }}} = 4A[V_{{CE\left( {\text{ max }} \right)}} I_{{o{ }}} \times D\left] { + 4B[I_{D} \times V_{DS} } \right]$$

15 $$PB_{{losses{ }}} = 4A[I_{D} \times V_{DS} \left] { + 4B[V_{{CE\left( {\text{ max }} \right)}} I_{{o{ }}} \times D} \right]$$

**Table 5:** Rating of the parameters for both groups of modules.

Parameters	Group-A IGBTs modules	Group-B MESFETs modules
Total Power of each group	$425 \;{\text{kW}}$	$75 \;{\text{kW}}$
Input power of each module in a particular group	$106.25 \;{\text{kW}}$	$18.75\;{\text{kW}}$
Total input voltages of each group	$9.35\;{\text{ kV}}$	$1.65 \;{\text{kV}}$
Input voltages of each module in a particular group	$2.34 \;{\text{kV}}$	$412.5 \;{\text{V}}$
Total input current each group	$45.45 \,{\text{A}}$	$45.45 \;{\text{A}}$
Input current of each module in a particular group	$45.45 \;{\text{A}}$	$45.45 \;{\text{A}}$
Total output voltages of each group	$440 \;{\text{V}}$	$440\;{\text{V}}$
Output voltages of each module in a particular group	$440 \;{\text{V}}$	$440\;{\text{V}}$
Total output current each group	$510 \;{\text{A}}$	$90 \;{\text{A}}$
The output current of each module in a particular group	$127.5\;{\text{ A}}$	$22.5\;{\text{ A}}$
Switching frequency	$10 \;{\text{kHz}}$	$200 \;{\text{kHz}}$

$PA_{{\text{losses }}}$ are losses by IGBTs converters relying on the wide bandgap material and $PB_{{losses{ }}}$ are losses by wide bandgap converter relaying on the MESFETs converter. System Parameters used in our proposed model are the data sheets of IKW25N120T2 (IGBT) and NES1823P-100 Datasheet (GaAs MESFET), then the power losses in $PA_{{\text{lo es }}}$ and $PB_{{\text{losses }}}$ are found $11.5\;{\text{kW}}$ and $1.5\;{\text{kW}},$ respectively. On the other hand, our proposed model, which hybrid modules of both types of converters, found $3.5{\text{ kW}}$. Then the efficiency can be calculated in^99^; we modified it accordingly as:

16 $${\upeta } = \frac{{P_{in} - P_{losses} }}{{Total{ }\;Input{ }\;power}} \times 100$$

Then, efficiencies we found from Eq. (16) are 98%, 99.4%, and 99.25% for the, $PandB_{{\text{losses }}}$ and our proposed model, respectively.

**Figure 12:**
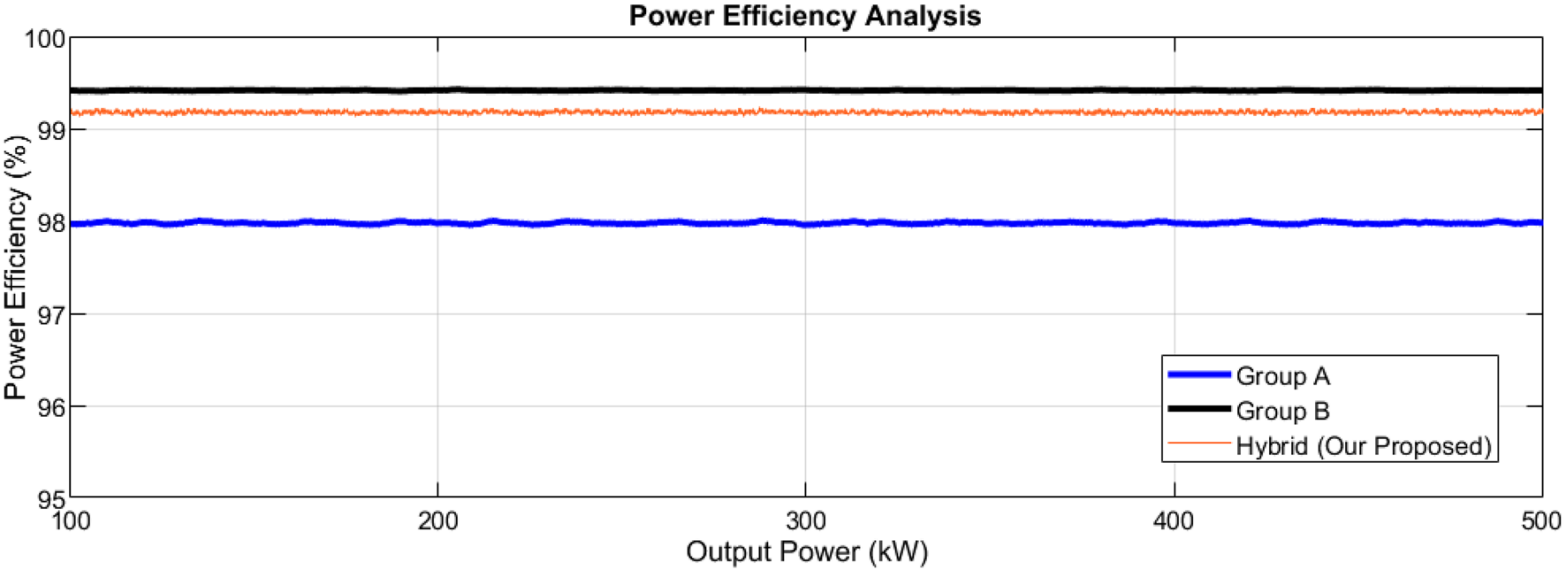
Outpower vs power efficiency curves analysis.

The analysis of the outpower vs power efficiency curves is shown in Fig. 12. The power density can be found after getting the power efficiencies and power losses. The power density is defined as the ratio of the out power and the total volume contained by switching circuits, transformer core/windings, and cooling chips. Then the equation becomes:

17 $${\uprho } = \frac{{P_{in} - P_{losses} }}{{total{ }\;volume{ }\;of{ }\;equipment}} \times 100$$

The total volume of the equipment used in the complete modules is estimated in^99^, which we considered the same for our calculations. The power density in the group of the $PA_{{\text{losses }}}$ is a little higher which contains 13.5 kW/L because of the conventional IGBTs group of modules. On the other hand, the power density in the group $PB_{{\text{losses }}}$ is small and contains 11.75 kW/L because of the conventional IGBT group of modules. In our proposed model, the power density is estimated to be 10.99 kW/L.

**Figure 13:**
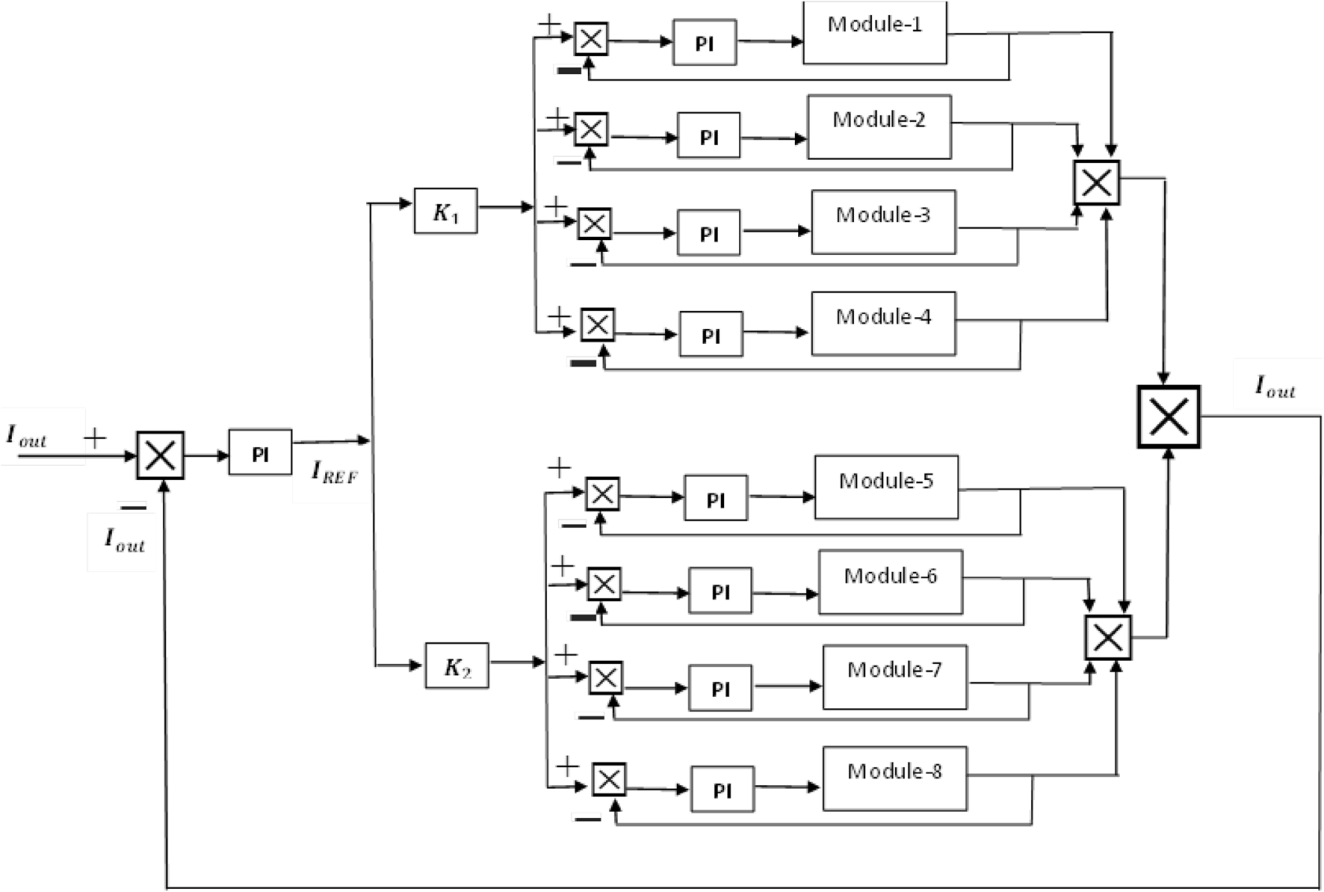
Output current sharing configuration for the proposed hybrid multimodule DC–DC Converter.

The modules of both groups are not identical; therefore, there is a need for a control strategy that can handle the issue of mismatching among electrical parameters. It can provide thermal stress on the devices and cause uniformity among the power-sharing of the modules. A control strategy is presented for the converters, which are connected in series on the input side and parallel on the output side. So, the input series and output parallel topology is proposed by providing cross feedback output sharing configuration as shown in Fig. 13. For even power distribution in this case; the combination input series output parallels converter is connected to the cross feedback current sharing unit. Table 3 has a list of the design specifications that are meted out by our proposed controller. The OCS controller, shown in Fig. 13, comprises one outside output current loop and eight inner current loops. The primary multimodule group receives four of the eight inner loops, while the secondary multimodule group receives the four remaining inner loops. The primary focus of this paper is providing fast charging, and this current control strategy is based on but charging and negative pulse charging. The fractions 17/20 and 3/20 are denoted by $K_{1} and K_{2}$ respectively. As there are different current and voltage ratings, there is a need for equal input voltage sharing (IVS) and equal output current sharing (OCS). Hence, the voltages of the first module have been reduced to $V_{inA} /17,$ and the current has reduced to $I_{inA} /17$. The terms $V_{inA}$, $V_{inB}$, $I_{inA}$, $I_{inB}$ are used for the voltages and current of the first and second groups, respectively, while these specifications are modeled in a small signal model, as shown in Fig. 11. As the switching frequency of Group-B modules is $200 Kz$ therefore, we need a fast-charging time with a lower temperature. The proposed control strategy first controls the output current profile based on reflex charging with the help of output current. In the current output profile, the charging rate should not be increased because it causes overcharging, and ultimately, it decreases the life of the battery. These output currents behave as an outer current loop and inner current loops, as shown in Fig. 13. the same mechanism is adapted further as the feedback current of the first module is its output current, and the same pattern is applied to modules 2–3. Similarly, the feedback current of the third module is its output current, as shown in Fig. 13 (second group of modules). In steady-state conditions, all feedback currents should face the common reference (w.r.t output current loop) with zero static error.

**Figure 14:**
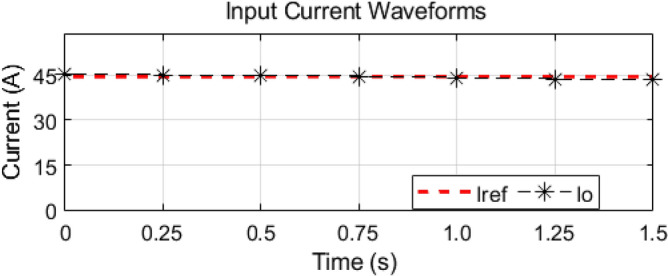
Input Current for the proposed hybrid 8-module DC–DC Converter.

**Figure 15:**
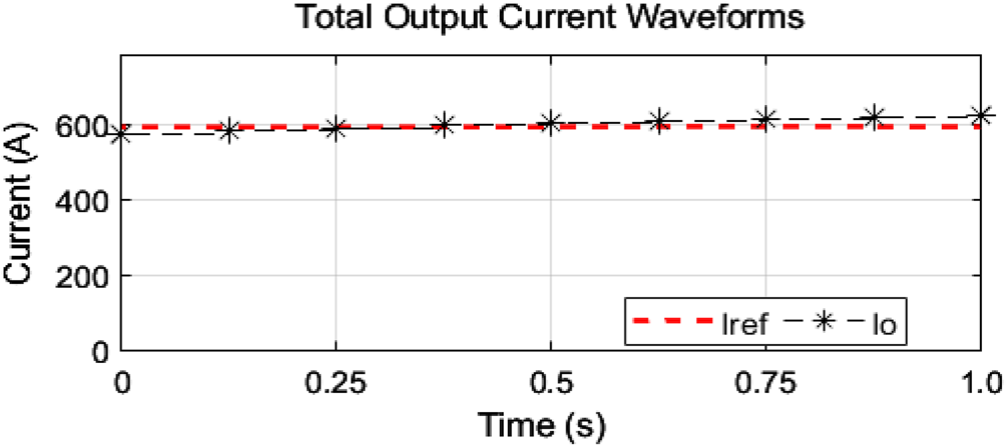
Total output Current for the proposed hybrid 8-module DC–DC Converter.

**Figure 16:**
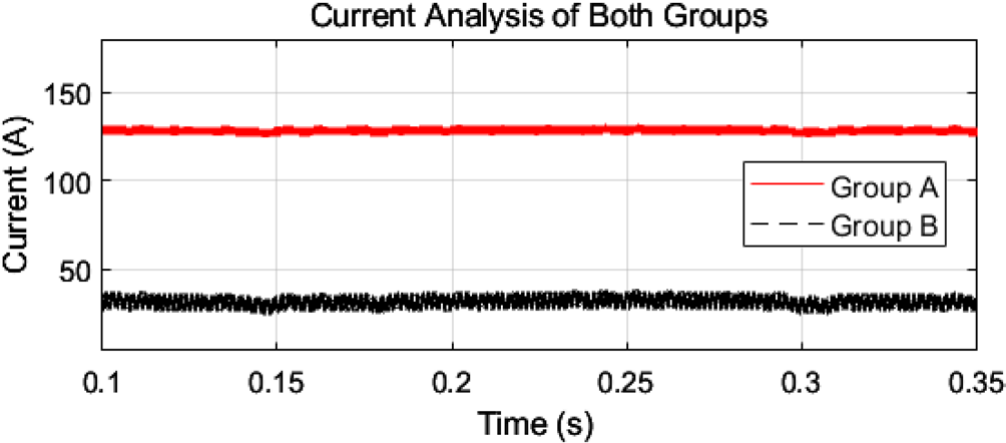
Output currents of both groups for the proposed hybrid 8-module DC–DC Converter.

**Figure 17:**
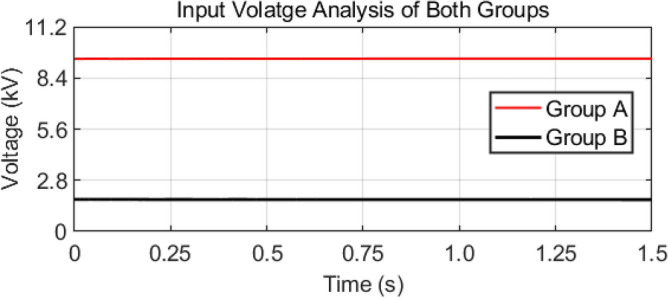
Input Voltages of both groups for the proposed hybrid 8-module DC–DC Converter.

**Figure 18:**
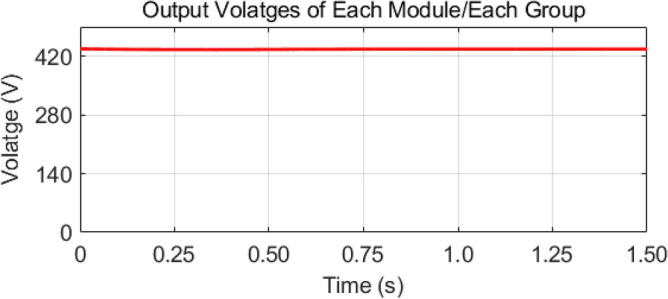
Output Voltages of Each Module/Each Group for the proposed hybrid 8-module DC–DC Converter.

**Figure 19:**
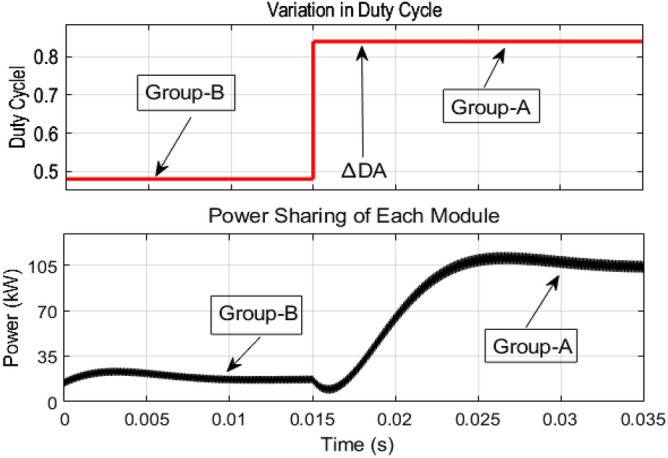
Power sharing analysis of both modules with variation in duty cycles.

**Figure 20:**
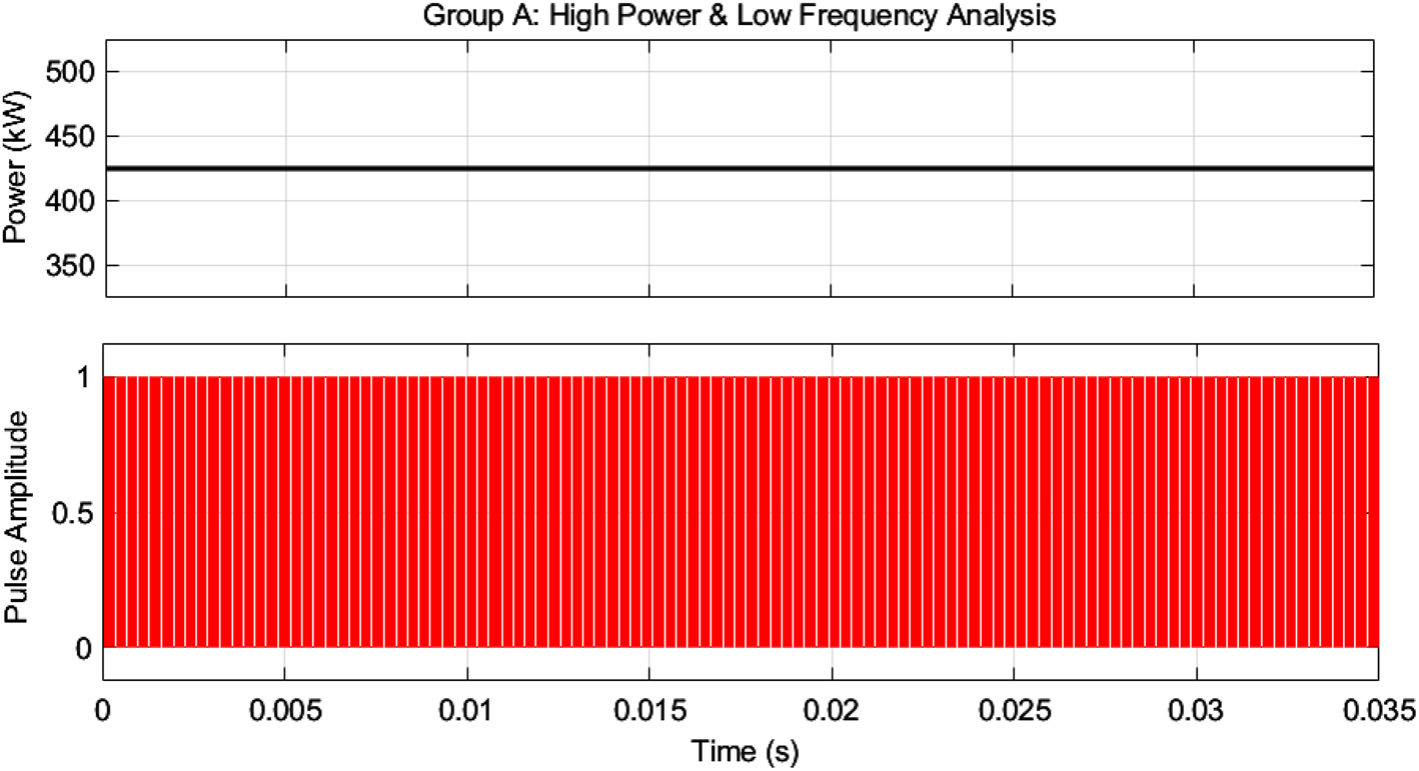
High power and low-frequency analysis for Group-A of IGBTs Modules.

**Figure 21:**
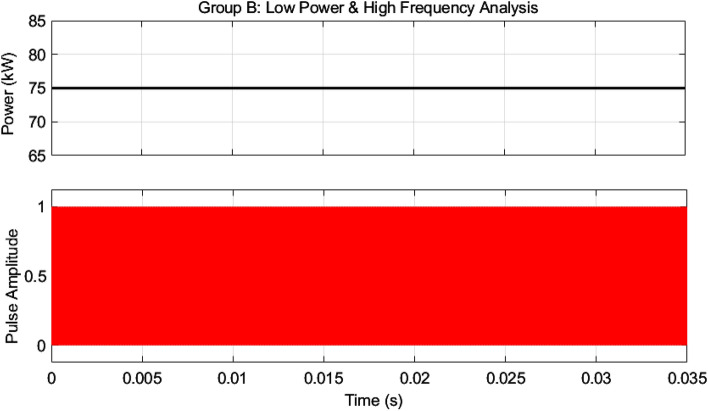
High power and low-frequency analysis for Group-B of MESFETs Modules.

**Figure 22:**
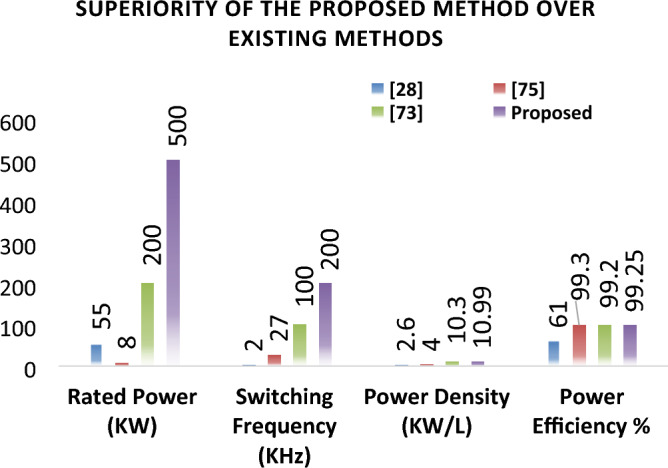
Superiority analysis of the proposed method over existing methods.

As mentioned earlier, the first group delivers high power and low frequency, whereas the second group caters to low power with high frequency. We are testing our simulation on the 440 V battery with an input power of 500 kW where the grid voltages are 11 kV. The first group extracts a high portion of the power, 85% of the total power, 500 kW, which is 425 kW. The second group deals with the lower portion of power, which is 15% of total power, which is 75 kW. The switching frequencies are proposed to be 10 kHz and 200 kHz for the first and second groups, respectively. So, the injected voltage from the grid to the first group is 17/20 of 11 kV, which is 9.35 kV, and the second group takes a voltage of 3/20 of 11 kV, which is 1.65 kV. So, the topology is based on the input series and output parallel, and the current in each module remains at the same current of 45.45 A, as shown in the Fig. 14 which ensures our ratting given in Table 3. On the other hand, the total output Current for the proposed hybrid 8-module DC–DC Converter, which is 600 A, is also tested with reference current and ensures uniformity, as shown in Fig. 15. The charging current required for the battery is 600 A, where the first group is responsible for 17/20 of 600 A, which is 510 A, and the second group can provide 3/20 of 600 A, which is 90 A. These 510 A and 90 A current for group A and Group B are further divided by four as each group contains four modules. So, per module, currents are 127.5 A and 22.5 A for group A and Group B, respectively in Fig. 16. To ensure the input voltages are different in both modules, we also examined the input voltages, as shown in Fig. 17. As mentioned earlier, we are testing our simulation on the 440 V battery, as shown in Fig. 18. We formulated the power-sharing concept in Table 2 and further modeled it in Eqs. 8–13, while the change in duty cycle has a major effect on power sharing among both modules. To keep this importance, we have examined the power-sharing analysis of both modules with variation in duty cycles in Fig. 19. The Duty cycle changes from 0.45 to 0.85 between power share of $106.25 \;{\text{Kw}}\;{\text{ and}}\; 18.75\;{\text{ Kw}}.$ As earlier discussed, two different modules of low frequency ($10 \;{\text{kHz}}$) with high power and high frequency $\left( {200 \;{\text{kHz}}} \right)$ with low power are considered. The analysis frequency and power are shown in Figs. 20 and 21 for Group-A of IGBTs Modules and Group-B of MESFETs Modules, respectively. Figure 22 highlights our contribution as compared to other works. In^28^, the work is old-fashioned as it uses a 2 kHz switching frequency. In^100^, WBG material was used and got 99.3% efficiency at a low scale and low switching frequency of 27 kHz. The model presented in^50^ has good power efficiency and density with a 100 kHz switching frequency. On the other hand, our proposed model has good power efficiency, high power density, and an ultra-high switching frequency of 200 kHz. The reason for this high switching is the development of MESFET switches in the second group of modules, and this makes our model efficient for others, as shown in Fig. 22.

## Conclusions and future research directions

This article presents a hybrid multimodule DC–DC converter based on DAB topology for EVs that are designed to increase power efficiency and power density. Eight modules from two different groups of semiconductor devices make it a hybrid concept, a multimodule DC–DC converter. The first group consists of four modules of IGBTs, which have a low switching frequency and a very high fraction of the total power. Similarly, the second group consists of four modules of MESFETs based on wide bandgap material, which has a high switching frequency with a lower fraction of power. Both groups of wideband devices can provide ultra-high switching frequencies, but these are not able to handle high power as the charging of the vehicle systems is required. The performance analysis of these semiconductor devices is done where the first group extracts a high portion of the power, which is 85% of the total power of 500 kW, and the second group extracts a lower portion of the power, which is 15% of the total power. The fast-charging phenomenon is achieved by providing switching frequencies of 10 kHz and 200 kHz for the first and second groups, respectively. WBG materials have the ability to switch fast, have high power efficiency, have high power density, and have low switching losses, but they can deal with high amounts of power. In this article, a generalized small-signal model is analyzed as a control strategy required to achieve uniform power sharing between the employed modules. An output current-sharing configuration ensures uniformity among individual modules where the current of the controller is compared and tested with a reference current. Simulation results on MATLAB show the confirmation of current uniformity and provide the charging current to the batteries of the EVS. The conduction losses of both groups are examined. We achieved a power efficiency of 99.25% and a power density of 10.99 kW/L, which is remarkable at 200 kHz fast switching. The future work can be extended by adding more advanced WBG devices that can handle a larger fraction of the power. Future research directions include exploring advanced wide bandgap devices such as GaN and SiC for enhancing converter performance, efficiency, and power density^101^. Novel control strategies, possibly based on artificial intelligence or machine learning algorithms, could improve converter stability and reliability, particularly in dynamic load conditions. Additionally, system integration and optimization of the hybrid DC–DC converter within the EV charging infrastructure need attention, along with understanding scalability and application flexibility for different power levels and grid constraints^102^. Finally, a comprehensive techno-economic analysis should be conducted to evaluate the cost, performance, and environmental impact of the hybrid converter compared to conventional solutions, informing its commercial viability in the growing electric vehicle market^103^.

## Data Availability

The datasets used and/or analysed during the current study available from the corresponding author on reasonable request.
